# *Cryptococcus gattii* VGIII Isolates Causing Infections in HIV/AIDS Patients in Southern California: Identification of the Local Environmental Source as Arboreal

**DOI:** 10.1371/journal.ppat.1004285

**Published:** 2014-08-21

**Authors:** Deborah J. Springer, R. Blake Billmyre, Elan E. Filler, Kerstin Voelz, Rhiannon Pursall, Piotr A. Mieczkowski, Robert A. Larsen, Fred S. Dietrich, Robin C. May, Scott G. Filler, Joseph Heitman

**Affiliations:** 1 Department of Molecular Genetics and Microbiology, Duke University Medical Center, Durham, North Carolina, United States of America; 2 David Geffen School of Medicine at UCLA, Division of Infectious Diseases, Los Angeles Biomedical Research Institute at Harbor-UCLA Medical Center, Los Angeles, California, United States of America; 3 Institute of Microbiology & Infection and the School of Biosciences, University of Birmingham, Birmingham, United Kingdom; 4 National Institute of Health Research Surgical Reconstruction and Microbiology Research Centre, Queen Elizabeth Hospital, Birmingham, United Kingdom; 5 Department of Genetics, University of North Carolina at Chapel Hill, Chapel Hill, North Carolina, United States of America; 6 Division of Infectious Diseases, Keck School of Medicine, University of Southern California, Los Angeles, California, United States of America; 7 Department of Medicine, Duke University Medical Center, Durham, North Carolina, United States of America; 8 Department of Pharmacology and Cancer Biology, Duke University Medical Center, Durham, North Carolina, United States of America; Ohio State University, United States of America

## Abstract

Ongoing *Cryptococcus gattii* outbreaks in the Western United States and Canada illustrate the impact of environmental reservoirs and both clonal and recombining propagation in driving emergence and expansion of microbial pathogens. *C. gattii* comprises four distinct molecular types: VGI, VGII, VGIII, and VGIV, with no evidence of nuclear genetic exchange, indicating these represent distinct species. *C. gattii* VGII isolates are causing the Pacific Northwest outbreak, whereas VGIII isolates frequently infect HIV/AIDS patients in Southern California. VGI, VGII, and VGIII have been isolated from patients and animals in the Western US, suggesting these molecular types occur in the environment. However, only two environmental isolates of *C. gattii* have ever been reported from California: CBS7750 (VGII) and WM161 (VGIII). The incongruence of frequent clinical presence and uncommon environmental isolation suggests an unknown *C. gattii* reservoir in California. Here we report frequent isolation of *C. gattii* VGIII *MAT*α and *MAT* **a** isolates and infrequent isolation of VGI *MAT*α from environmental sources in Southern California. VGIII isolates were obtained from soil debris associated with tree species not previously reported as hosts from sites near residences of infected patients. These isolates are fertile under laboratory conditions, produce abundant spores, and are part of both locally and more distantly recombining populations. MLST and whole genome sequence analysis provide compelling evidence that these environmental isolates are the source of human infections. Isolates displayed wide-ranging virulence in macrophage and animal models. When clinical and environmental isolates with indistinguishable MLST profiles were compared, environmental isolates were less virulent. Taken together, our studies reveal an environmental source and risk of *C. gattii* to HIV/AIDS patients with implications for the >1,000,000 cryptococcal infections occurring annually for which the causative isolate is rarely assigned species status. Thus, the *C. gattii* global health burden could be more substantial than currently appreciated.

## Author Summary

The environmentally-acquired human pathogen *C. gattii* is responsible for ongoing and expanding outbreaks in the Western United States and Canada. *C. gattii* comprises four distinct molecular types: VGI, VGII, VGIII, and VGIV. Molecular types VGI, VGII, and VGIII have been isolated from patients and animals throughout the Western US. The Pacific Northwest and Canadian outbreak is primarily caused by *C. gattii* VGII. VGIII is responsible for ongoing infections in HIV/AIDS patients in Southern California. However, only two environmental *C. gattii* isolates have ever been identified from the Californian environment: CBS7750 (VGII) and WM161 (VGIII). We sought to collect environmental samples from areas that had confirmed reports of clinical or veterinary infections. Here we report the isolation of *C. gattii* VGI and VGIII from environmental soil and tree samples. *C. gattii* isolates were obtained from three novel tree species: Canary Island pine, American sweetgum, and a Pohutukawa tree. Genetic analysis provides robust evidence that these environmental isolates are the source of human infections.

## Introduction

Outbreaks of infectious diseases caused by all major classes of microbial pathogens occur globally, annually, and these outbreaks pose public health challenges [1]–[4]. Advancing understanding of forces driving outbreaks to enhance our ability to predict, contain, and blunt their impact include: 1) identification of environmental sources and vectors, and 2) defining genetic mechanisms that give rise to infectious microbes with altered virulence or transmissibility.

Over 200 species of fungi are recognized as human/animal pathogens [5]. Fungal pathogens also cause outbreaks of disease. This includes clusters of infections caused by *Coccidioides immitis/posadasii*, *Histoplasma capsulatum*, or *Apophysomyces trapeziformis* following soil perturbations (earthquakes, tornadoes, dust storms, construction, landscaping) [6]–[13]. The *Cryptococcus* pathogenic species complex includes the globally distributed human pathogens *C. neoformans* and *C. gattii*, which cause significant fungal disease burden and increasing public health costs in immunocompromised and immunocompetent individuals worldwide [14]–[19]. *Cryptococcus* is annually responsible for >1,000,000 infections, >620,000 deaths, and one-third of all AIDS related deaths [17]. *Cryptococcus neoformans* and *C. gattii* have been isolated from various environmental sources (soil, trees, bird guano) but reports genetically linking specific environmental reservoirs to individual cases are limited because: 1) infections often take considerable time to diagnose, 2) are not reportable diseases in the USA and abroad, 3) *C. neoformans* and *C. gattii* are not often distinguished by species or by molecular type, and 4) retrospective environmental surveys are rare and scattered, may lack molecular analysis and corresponding clinical isolates, and/or follow many years after reported clinical infections [13], [20].

*Cryptococcus gattii* comprises four distinct molecular types: VGI, VGII, VGIII, and VGIV, without evidence of genetic exchange of nuclear genomes, providing evidence the four molecular types represent distinct, related species [16], [21]–[25]. *C. gattii* VGII and VGIII are associated with two distinct expanding outbreaks in the Western US [22], [26]. Phylogenetic analysis suggests that *C. gattii* and *C. neoformans* diverged ∼40 million years ago and VGII is the ancestral molecular type to the *C. gattii* clade [23], [24], [27]–[30]. Genetic rearrangement between the VG types could actively suppress recombination, acting as reproductive barriers, and limiting productive recombination. However, analysis of the mitochondrial genomes of *C. gattii* VGI and VGII lineages indicates a highly clonal mitochondrial genome within each lineage consistent with uniparental mitochondrial inheritance but different genealogies support the hypothesis that examples of mitochondrial genome transmission from VGII into VGI isolates have occurred [31], [32]. Furthermore, transmission of hypervirulence traits within and between different molecular types was recently demonstrated in laboratory crosses [33]. Thus, given evidence linking mitochondrial function to enhanced intracellular proliferation of VGII outbreak isolates in macrophages, and previous studies linking mitochondria to virulence of plant fungal pathogens, these findings illustrate how genetic exchange can impact virulence of pathogenic fungi [33]–[35].

*C. gattii* molecular type VGII, which is highly virulent and has a predilection for infecting apparently healthy hosts, is causing an outbreak on Vancouver Island that has expanded to the Canadian mainland and also Washington, Oregon, and possibly California [21], [22], [36], [37]. As a result of increased sampling and molecular analysis, three sub-molecular types are now recognized: VGIIa, VGIIb, and VGIIc. The Vancouver, BC and Pacific Northwest (PNW) outbreak is characterized by infections caused predominantly by VGIIa, the major genotype, with a lower frequency of incidence of the VGIIb minor genotype. A novel, distinct, highly virulent molecular type designated as VGIIc is presently restricted to Oregon [26], [38]. Prior to the now recognized outbreak in the Pacific Northwest, *C. gattii* VGIIa was isolated in 1970 from a human sputum sample (Seattle, Washington, NIH444) and in 1992 a closely related VGII isolate was obtained from an environmental sample from San Francisco, California (CBS7750) [21], [39], [40]. Sporadic isolates possibly related to the PNW VGIIa major genotype at a limited number of MLST loci have been reported outside the USA [25], [41], [42]. Due to the scarcity of these VGIIa related genotypes outside the USA, the environmental reservoir and source has not been established. On the other hand, while isolates related to the VGIIb minor genotype at a limited number of MLST loci have been reported from several geographical regions, VGIIb isolates that are indistinguishable across 30 MLST loci have only been reported from Australia, thus providing robust evidence that this is the likely geographic source of the VGIIb outbreak isolates [21], [25], [27], [42], [43].

In contrast, *C. gattii* molecular type VGIII is responsible for ongoing infections in immunocompromised HIV/AIDS patients in Southern California and the Southwestern US [18], [22], [37], [44], [45]. Outside the US VGIII has been associated with sporadic infections in Brazil, Colombia, Mexico, India, Germany, and Korea [14], [23], [37], [46]–[51]. Despite the high preponderance of infections caused by *C. gattii* VGIII in the HIV/AIDS population of California and the Southwestern USA, only one environmental VGIII isolate (WM161) has ever been recovered from California [23], [37], [44], [52]. On the other hand, in Colombia and Argentina VGIII isolates have been isolated from the environment, yet clinical prevalence appears low in those localities [47], [53]–[55].

*C. gattii* VGIII has been further classified into two groups, VGIIIa and VGIIIb, based on MLST analysis [22]. Analysis of the Californian VGIII clinical population indicated that VGIIIa is more clonal with only one identified *MAT* **a** isolate in comparison to VGIIIb, in which both *MAT*α and *MAT* **a** isolates were frequently identified [22], [45]. Unlike other VG molecular types or *C. neoformans*, *MAT* **a** isolates are frequently isolated from both clinical and environmental populations of VGIIIb, suggesting that the VGIIIb population may be fertile and actively undergoing **a**-α sexual recombination in nature [56]–[58]. VGIII environmental isolates have been reported from *Tipuana tipu* trees in Argentina, *Terminalia catappa*, *Corymbia ficifolia*, *Eucalyptus sp*, and *Ficus sp.* in Colombia, and *Manilkara hexandra* in India [47], [49], [53]–[55]. Analysis of 60 VGIII isolates from California resulted in the identification of only four alleles shared between VGIIIa and VGIIIb in four independent isolates, *CAP10* and *TEF1* appear to be ancestral, while the shared *PLB1* allele appears to have been introgressed between VGIIIa and VGIIIb [22]. The MLST analysis, haplotype analysis, paired allele graphs, percentages of compatible loci, and indices of association all support the divergence of the VGIIIa and VGIIIb subtypes, limited recombination within VGIIIa, and more frequent recombination in VGIIIb [22].

Prior to the Pacific Northwest outbreak caused by *C. gattii* VGII, California was historically recognized as the oldest known region associated with the occurrence of cryptococcosis within the USA. Early studies denoted California as the most prominent site of serotype C infections, later identified as *C. gattii* VGIII. Byrnes et al. 2011 was the first to establish the prevalence of *C. gattii* VGIII within the HIV/AIDS clinical population of California but did not obtain nor identify any VGIII isolates directly from the environment [22]. Of note, the MLST profile of the historically identified environmental isolate WM161 did not match the MLST profiles of any of the reported clinical isolates obtained from California; therefore, the infectious environmental reservoir of *C. gattii* VGIII remained unknown. Previous environmental surveys within the Pacific Northwest, USA have identified the environmental niche of *C. gattii* VGII [59], [60]. The lack of genetic exchange between the molecular types, genomic rearrangements confined within molecular types, and differences in host predilection strongly suggest that the molecular types recognized within *C. gattii* comprise cryptic species. We therefore sought to identify the environmental reservoir of *C. gattii* VGIII in California by sampling plants and soil in areas near residents with confirmed *C. gattii* infections.

As a result of this study we have identified the local environmental source of *C. gattii* VGI and VGIII associated with trees and soil debris in the greater Los Angeles area of California. Molecular typing and whole genome analysis of the Californian *C. gattii* VGIII environmental isolates indicates a high amount of molecular diversity exists in this region, VGIIIa and VGIIIb can colonize similar and overlapping niches, and some environmental isolates are closely related to clinical isolates and the likely source of previously reported human infections, and a potential reservoir for inciting new infections. Furthermore, our results suggest that many of these isolates are sexually competent and capable of intra- and inter-molecular mating which might facilitate dispersal and propagate altered infectious characteristics such as virulence or transmissibility. Further work is needed to fully elucidate the links between *Cryptococcus* molecular types, environmental distributions, and the propensity to initiate disease in exposed hosts.

## Results

### Identification of the environmental source of *C. gattii* VGIII in Southern California

**Figure 1:**
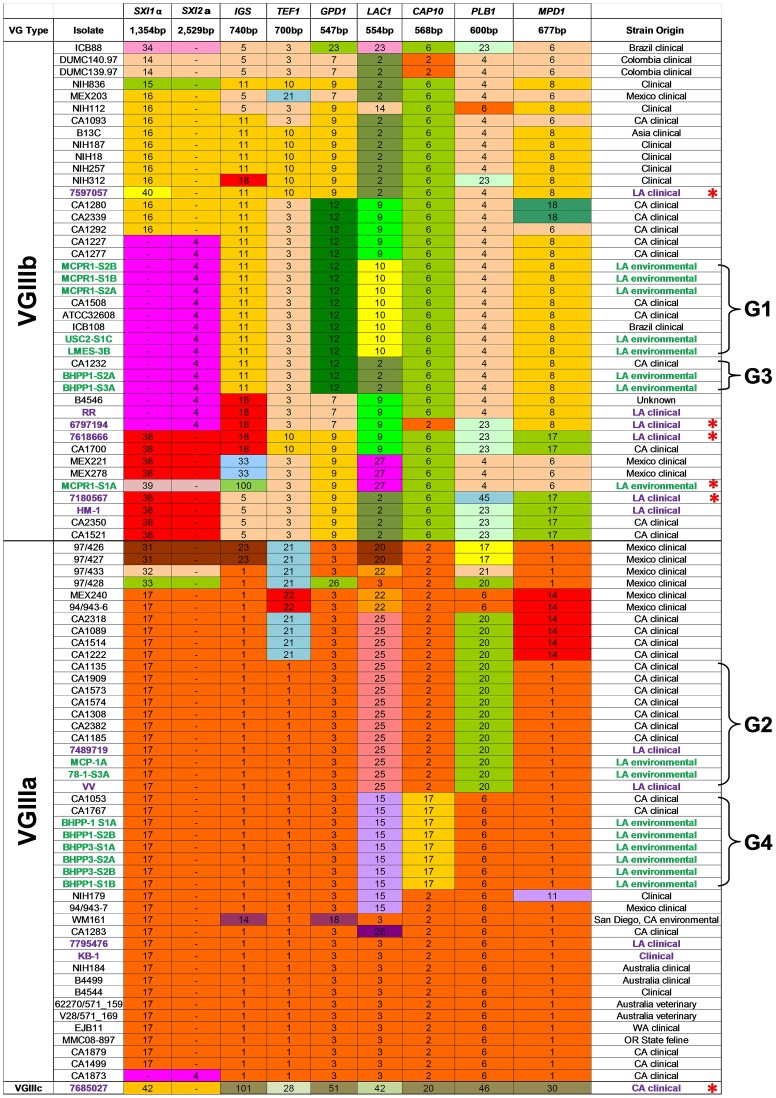
Global molecular analysis illustrates a diverse population of *C. gattii* VGIII isolates are endemic to Southern California. Multilocus sequence typing was performed on 8 loci. Unique alleles were assigned a distinct color for each marker. New environmental (green, n = 13) and clinical (Purple, n = 5) VGIII isolates from California are compared to previously analyzed VGIII isolates (Table adapted from [22]). Seven VGIIIa and five VGIIIb environmental isolates representing four MLST groups shared indistinguishable MLST profiles with previously reported clinical isolates as indicated (brackets, denoted as Groups 1–4 (G1, G2, G3, and G4). New MLST sequence types were observed for both VGIIIa (n = 3) and VGIIIb (n = 3) and are denoted with red asterisks.

The long term and extensive clinical prevalence of *C. gattii* in California has implicated a local endogenous reservoir but even with increased environmental sampling the environmental source of infections has remained undefined. We therefore sought to collect environmental samples from areas that had confirmed reports of clinical and/or veterinary infections. From soil and swab samples collected at 24 locations throughout the greater Los Angeles area over a two year period (Figure S1), we screened 146 environmental (n = 107) and clinical (n = 39) isolates utilizing canavanine glycine bromothymol blue (CGB) and niger seed (NGS) indicator media. We identified 30 (20.6%) potential *C. gattii* isolates, 11 clinical and 19 environmental isolates obtained from 4 out of 24 sites (16.7%) that were melanin positive on NGS and produced a blue color reaction on CGB agar (Figure 1 and Table S4). Eleven of 39 clinical (28.2%) and 19 of 107 (17.8%) environmental isolates were also confirmed as *C. gattii* via differences in *ATP6* PCR product length and MLST analysis (Figure 1, Tables S4 and 5) [61]. *C. gattii* was isolated in association with three novel host tree species: *Pinus canariensis* (Canary Island pine, isolates LMES-3A, MCP-1A, MCPR1-X, USC-X, USC2-SIC), *Liquidamar styraciflua* (American sweetgum, isolates 78-1-S3A, BHPP3-X), and *Metrosideros excels* (Pohutukawa tree, isolate BHPP1-X). All *C. gattii* isolates were haploid by FACS analysis (Table S4). An additional 100 isolates were further identified as *C. neoformans* based on positive pigmentation on NGS agar, negative blue color formation/no growth on CGB agar, and *IGS1* sequence (data not shown).

### MLST analysis indicates a diverse population of *C. gattii* VGIII are endemic to Southern California

To characterize the molecular types prevalent in the Californian isolates, MLST analysis was performed on 11 unlinked loci for the 11 clinical and 19 environmental *C. gattii* strains obtained from Los Angeles, California USA. The data were compared to previously reported VGIII isolates [22] to determine the presence of novel or shared MLST profiles between environmental and clinical isolates (Figure 1 and Table S4). Isolates primarily clustered into three previously recognized molecular types: VGI, VGIIIa, and VGIIIb. The VGI molecular type found in California shares the most common MLST type observed worldwide (Table S5). Both VGIIIb *MAT*α (5/14, 36%) and *MAT* **a** (9/14, 64%) mating types were obtained from clinical and environmental isolates, suggesting the potential for an actively sexually recombining population in Southern California within this molecular type subgroup. In contrast, all of the VGIIIa isolates characterized were found to be *MAT*α, suggesting that opposite sex mating may be rare among isolates of this subtype. Overall, nine isolates typed as VGIIIb *MAT* **a** (2 clinical and 7 environmental), five as VGIIIb *MAT*α (4 clinical and 1 environmental), 12 as VGIIIa *MAT*α (4 clinical and 8 environmental), and three isolates typed as VGI *MAT*α (Tables S4 and S5). These results reveal a, widespread reservoir of *C. gattii* in the greater Los Angeles area as the likely source of frequent cryptococcal infections in HIV/AIDS patients in Southern California.

Sixteen isolates, 14 environmental (7 VGIIIa and 7 VGIIIb) and 2 clinical (1 VGIIIa and 1 VGIIIb), shared four different MLST profiles (designated groups G1 to G4, represented with brackets) with previously reported clinical isolates (Figure 1), indicating shared descent between recently identified environmental isolates and previously reported clinical isolates spanning an isolation period of up to 12 years. Six new MLST sequence types were identified: five VGIIIb (4 clinical and 1 environmental) and one uncategorized VGIII type (7685027) (Figure 1). Additional analysis of the *CAP59*, *TOR1*, *SOD1*, and *URA5* loci did not improve resolution of isolates that shared an indistinguishable 8-locus MLST profile. Thus, there was a high level of diversity in these environmental isolates reflected by the recovery of a multitude of distinct MLST profiles (Figure 1 and Table S4). Furthermore, we obtained both VGIIIa and VGIIIb molecular sub-types from the same sample (BHPP1 designation) or environmental location (MCP/MCPR or BHPP1/BHPP3 designations) suggesting that both molecular types can colonize the same ecological niche in California fostering the potential for hybridization. Thus, their lack of frequent genetic exchange suggests that the two molecular types could represent distinct species in which mating is limited by genetic and/or temporal barriers limiting productive introgressions between the molecular types.

**Figure 2:**
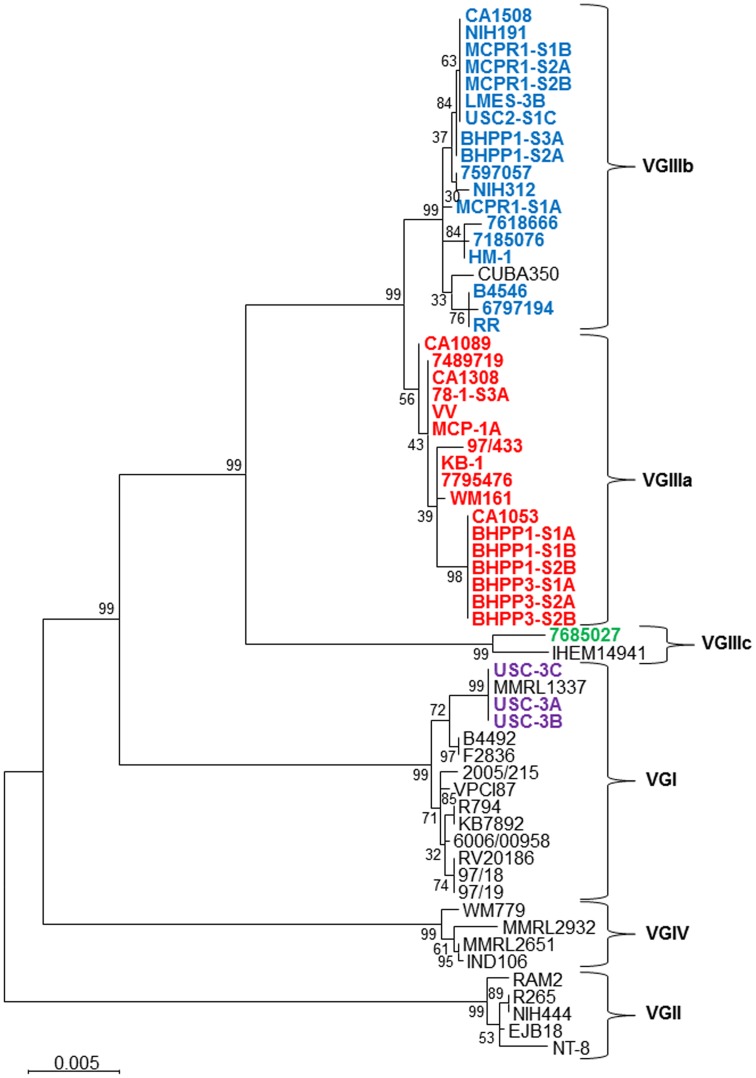
Phylogenetic analysis of newly identified *C. gattii* isolates from California. Phylogenetic tree obtained by analysis of 7 concatenated MLST loci (*IGS1*, *TEF1*, *GPD1*, *LAC1*, *CAP10*, *PLB1*, and *MPD1*) using 500 bootstrap replicates and the Maximum Likelihood (MLC) method based on Tamura-Nei model applying Neighbor-Join and BioNL algorithms. The tree with the highest log likelihood is shown. The analysis involved 61 sequences and a total of 4252 positions. Scale bar indicated 0.005 substitutions per nucleotide.

One clinical isolate (7685027) did not align with either of the two previously recognized VGIIIa or VGIIIb molecular types and either represents a novel VGIII subgroup that has been undersampled or a novel hypermutator isolate [62]. This isolate contains many completely novel MLST alleles (*URA5*, *TOR1*, *PLB1*, *MPD1*, *LAC1*, *CAP10*, *GPD1*, and *IGS1*) not observed previously (Figures 1 and 2), consistent with a possible origin via action of a hypermutator phenotype strain resulting in the rapid emergence of novel MLST alleles [62]. On the other hand, other MLST alleles (*SOD1*, *CAP59*, and *TEF1*) are shared with two additional isolates (IHEM14941S and IHEM14941W) obtained from a Mexican immigrant diagnosed in Spain and thus they may represent a novel VGIIIc sub-molecular type.

In addition to MLST analysis of nuclear loci, we expanded our analysis to explore the mitochondrial genome because the mitochondria are primarily uniparentally inherited from the *MAT* **a** parent, uniparental/biparental mitochondrial inheritance rates may differ between molecular types, and their genome evolves independently from the nuclear genome [32], [33], [63]–[66].

Phylogenetic analysis indicated that the MtLrRNA locus was indistinguishable between VGIIIa and VGIIIb isolates, suggesting closely related shared ancestry (data not shown). Utilizing *ATP6*-Byrnes primers, we observed distinct PCR size polymorphisms between VGI (300 bp), VGII (200 bp), and VGIII (≥600 bp) [61]. Additional PCR and MLST analysis of all VGIII isolates indicated a distinct size polymorphism between VGIIIa (600 bp) and VGIIIb (700 bp) except for a small subset of *C. gattii* VGIIIb isolates that exhibited a PCR product equivalent to the VGIIIa (600 bp) isolates. Large differences between the *ATP6*-Byrnes [33], [61] PCR product size limited informative phylogenetic sequence analysis between *C. gattii* molecular types so we utilized additional *ATP6*-Bovers primers previously used for mitochondrial analysis in *Cryptococcus* species [32]. Phylogenetic analysis of *ATP6*-Bovers primer sequences indicated two clades mostly segregating VGIIIa from VGIIIb, and *MAT* **a** from *MAT*α isolates as expected with the exception of a small group of clinical VGIIIb isolates (71805076, DUMC140.97, and HM-1) that share mitochondrial sequences with VGIIIa (Figure S2a). DUMC140.97 also shares nuclear sequence of *CAP10* (allele 2) with VGIIIb but no evidence of shared nuclear sequence is evident in the MLST analysis of HM-1 and 7180567. Haplotype network analysis indicates the *MAT* **a** VGIIIb allele as ancestral and the shared *MAT*α allele as derived suggesting further evidence of a rare introgression event with mitochondrial recombination occurring between VGIIIa and VGIIIb isolates (Figure S2b).

### Haplotype network analysis indicates rare introgression events between VGIIIa and VGIIIb molecular groups

**Figure 3:**
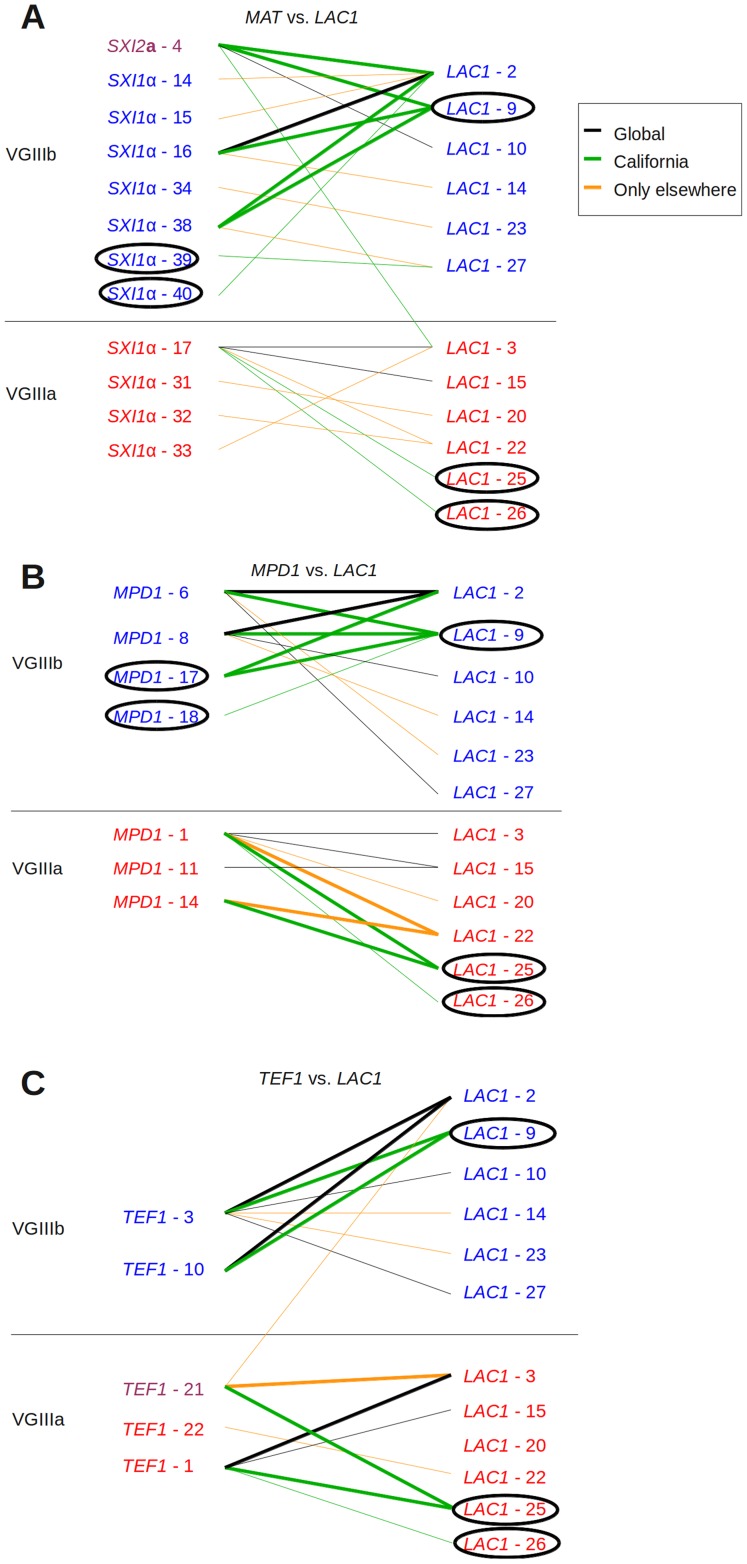
Evidence for recombination in Californian VGIII isolates. Informative paired allele diagrams depicting MLST data are presented. An hourglass shape indicates the presence of all four allele combinations, providing evidence for recombination. Alleles unique to California are circled, while black lines indicate combinations found both in Californian isolates and those from elsewhere. Green lines indicate combinations found only in California, and orange lines indicate combinations only found outside of California. (A) *MAT* versus *LAC1* paired allele analysis provides an example of recombination within the VGIIIb group in California, as well as one including one allele combination found additionally in Brazil and four in California. (B) *MPD1* versus *LAC1* and (C) *TEF1* versus *LAC1* provide examples of recombination within the VGIIIb group (top) and within VGIIIa (bottom). Additional informative comparisons are presented in Figure S3.

We further examined the presence and distribution of shared MLST alleles between VGIIIa and VGIIIb by paired allele and haplotype network analysis, determining the evolutionary history of each allele and testing for evidence of recombination within or between molecular types in California. Two clinical VGIIIb isolates (6797194 and 7618666) share *CAP10* allele 2 with known VGIIIa isolates. Paired allele diagrams between the *MAT* locus and *LAC1*, or *LAC1* and *TEF1*, illustrate recombination occurring in the Californian VGIII population (Figure 3). Evidence of recombination is present within VGIIIb and the more clonal VGIIIa population (Figure S3). Especially notable is the evidence for recombination between the *MAT* locus and *LAC1* in VGIIIb within the Californian isolates, suggesting that **a**-α mating is ongoing between isolates harboring *SXI2* **a** allele 4 and *SXI1*α allele 48.

**Figure 4:**
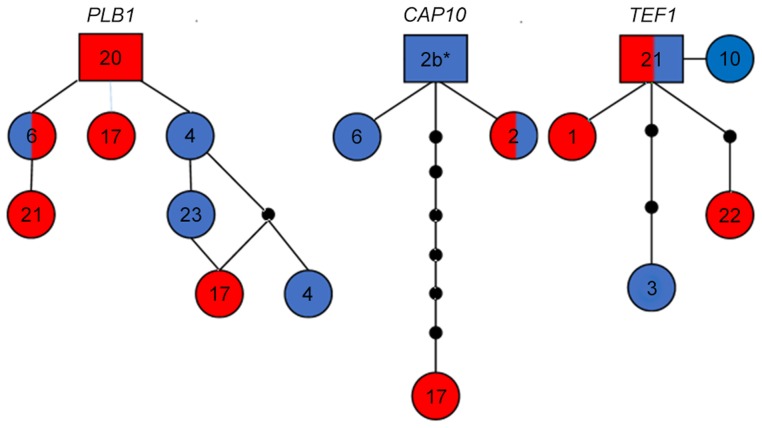
Haplotype network analysis suggests recent introgression between VGIIIa and VGIIIb. Alleles for each respective locus are indicated numerically. Alleles are colored red representing VGIIIa, blue representing VGIIIb, and dual-coloration representing alleles shared between the VGIIIa and VGIIIb molecular types. Alleles in squares represent the proposed ancestral allele, circles represent alleles present in the population, lines between alleles represent one predicted evolutionary event, and smaller black circles represent alleles that have not been recovered, or which may no longer be represented in the population. Haplotype networks for *PLB1* and *CAP10* exemplify a derived non-ancestral origin of alleles shared between the VGIIIa and VGIIIb populations. *CAP10* Allele 2b* (6797194) is represented as allele 2 in Figure 2 2 because over the reported and defined 611 bp the sequence identically matches. But from additional extended sequence reads of approximately 50 bp we observe a 1 bp substitution (T→C) at 23 bp downstream of the current reported *CAP10* allele sequence that distinguishes this allele from the standard allele 2. The *TEF1* haplotype network demonstrates ancestral origins of the shared allele 21.

Haplotype network analysis indicates that for two loci, *PLB1* and *CAP10*, the alleles shared between VGIIIa and VGIIIb isolates represent recent introgression events because the shared allele occupies a position other than the ancestral haplotype network allele (represented within the rectangle, Figure 4). In contrast haplotype network analysis of the *TEF1* share allele 21 represents the origin of the allele as ancestral. Additional haplotype network analysis of the evolutionary history of 9 additional MLST loci not shared between VGIIIa and VGIIIb robustly supports genetic isolation and provides evidence for cryptic speciation although evidence of additional shared alleles may emerge in the VGIII population with increased sampling and analysis of a broader set of nuclear loci (Figure S4).

### Whole genome sequences of MLST matched isolates indicates that environmental *C. gattii* isolates were the likely reservoirs associated with historical clinical infections

**Figure 5:**
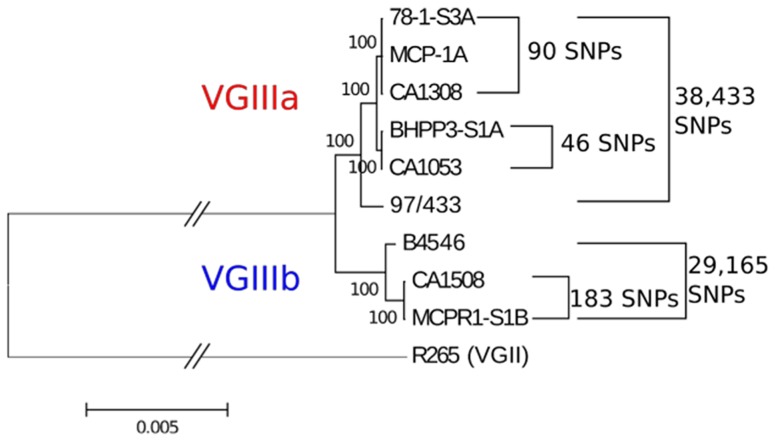
MLST matched clinical and environmental isolates are highly related. Maximum likelihood tree generated from a reference-based alignment to bases 310,000–410,000 from Supercontig 5 of VGII isolate R265 using 500 bootstrap replicates. Branch length has been truncated, and this is marked with hashes between the outgroup and cluster of VGIII isolates to improve readability. Scale bar indicates 0.005 substitutions per nucleotide position over this window of Supercontig 5. Total numbers of polymorphic sites differentiating each sub-clade of VGIII across the entire genome are indicated to the right of the tree.

We next determined that the newly isolated environmental strains are extremely closely related to the previously reported clinical strains based on whole genome sequencing. We sequenced matched clinical and environmental isolates representing 3 of 4 matched MLST groups, VGIIIa (group 1, CA1308, MCP-1A, 78-1-S3A and group 2, CA1053 and BHPP3-S1A) and VGIIIb (group 3, CA1508 and MCPR1-S1B) to determine whether environmental isolates could represent the source population for previously reported clinical infections. Whole genome sequence analysis of MLST matched clinical and environmental strains indicates that the genomes of grouped isolates are remarkably similar and very closely related (Figure 5 and Table S6, S7, and S8). The genomic sequences for these strains were aligned to the closest reference genome available, the VGII outbreak strain R265. SNP calling indicated a very high level of diversity in the total set of sequenced strains with a total of 773,900 polymorphic sites relative to the VGII isolate R265 [67]. This corresponds to approximately 4.4% of the genome. Within the VGIII sequenced set, there was ∼10-fold less diversity with 87,219 polymorphic sites corresponding to 0.49% of the ∼20 MB genome, which is still substantial. However, in spite of this diversity, the MLST matched isolates were remarkably similar to each other, ranging from a minimum of 46 differentiating sites (0.00026%) between VGIIIa isolates CA1053 (clinical) and BHPP3-S1A (environmental) to a maximum of 183 (0.0010%) between VGIIIb isolates CA1508 (clinical) and MCPR1-S1B (environmental, Figure 5). The SNPs identified within each matched set were characterized based on potential impact and the findings are summarized in Tables S6, S7, and S8. Variants altered three splice sites and introduced a moderate number of nonsynonymous SNPs. These nonsynonymous sites were also characterized based on predicted function, and a number of candidate pathogenesis factors were identified, including some with putative roles in heat tolerance, oxidative stress resistance, and stress response. Whole genome sequencing analysis indicates that the three identified environmental *C. gattii* populations are the likely environmental reservoirs for recent clinical infections and could serve as the source of additional infections.

### Environmental *C. gattii* VGIII isolates are fertile

**Figure 6:**
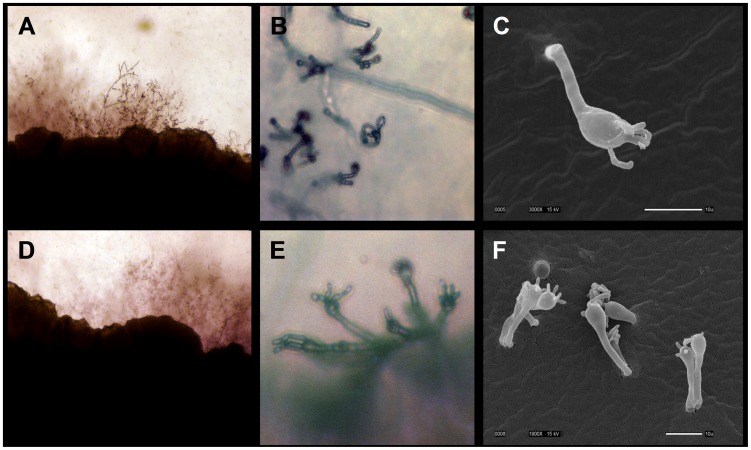
Environmental *C. gattii* isolates are fertile. (A, B, C) Isolate MCP-1A or (D, E, F) 78-1 S3A was mated with VGIII B4565**a** tester strain on V8 agar pH = 5 for 14 days at room temperature in the dark. (A, D) Hyphal growth along the mating patch edges is illustrated (100×). (B, E) Prolific basidia with chains of basidiospores are illustrated (400×). (C, F) Scanning electron micrographs show basidia and spores formed during sexual reproduction (Scale bar = 10 µm).

In addition to MLST analysis and population analysis, mating assays were conducted that established these environmental and clinical VGIII isolates are fertile with *C. neoformans* (VNI, VNII) and *C. gattii* (VGII, VGIII) isolates (Table S9a and S9b). Both *MAT* **a** and *MAT*α isolates produced positive mating reactions as assessed by light and electron microscopy with lateral hypha emanating from the colony (Figure 6), fused clamp connections (Figure S5a and S5b), basidia, and basidiospores (Figure 6 and Figure S5). All strains tested with the exception of two clinical strains (7618666 and 7795476) were fertile with at least one tester strain, although this did not always include mating with a VGIII strain (Table S7b). Both VGIIIb *MAT*α and *MAT* **a** isolates were fertile, and all VGIIIa *MAT*α environmental isolates were fertile with VNI, VNII, or VGIII tester strains (Table S9b). Environmental isolates were generally more fertile than clinical isolates; however, spore viability was not assessed to determine if these matings produced viable progeny. Clinical isolates are in general less fertile and may have undergone genetic or epigenetic modifications in the host that reduce their fertility. These results suggest that 1) VGIIIb isolates may undergo **a**-α opposite-sex mating in the environment, 2) many VGIIIa isolates are fertile and could participate in opposite-sex mating when a fertile *MAT* ***a*** isolate is present, and 3) interactions between molecular types could play a role in mating in the environment.

### Intracellular proliferation and mitochondrial tubularization appear decoupled in VGIII isolates

**Figure 7:**
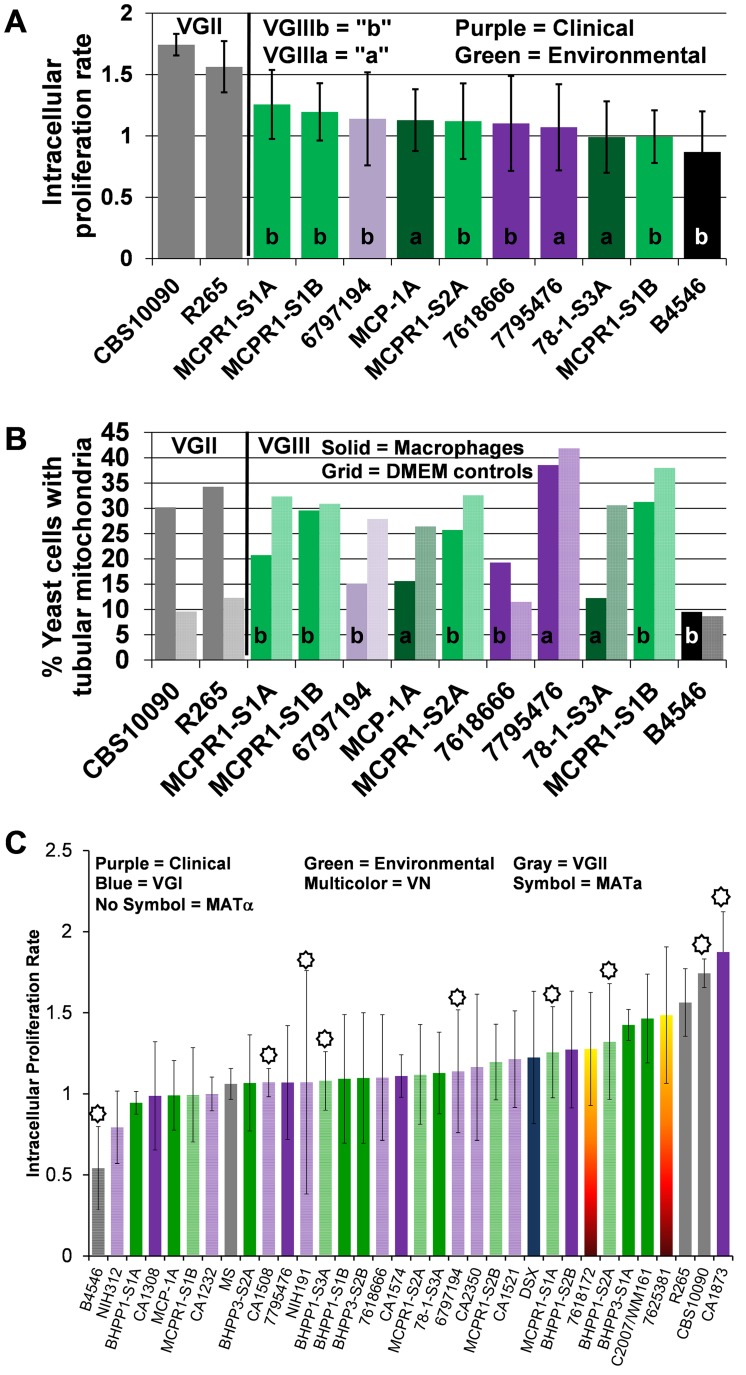
Intracellular proliferation rates (IPR) illustrate moderate proliferation levels and suggest mitochondrial tubularization is decoupled from IPR in macrophages for *C. gattii* VGIII isolates. (A) Intracellular *C. gattii* proliferation rates in macrophages were similar between environmental and clinical isolates. (B) Percent tubular mitochondria as a result of co-incubation with macrophages (solid bars) in comparison to DMEM controls (hatched bars). Percent tubular mitochondria does not correlate with IPR or virulence. Clinical isolates (VGIIIa dark-purple; VGIIIb light-purple), environmental isolates (VGIIIa dark-green; VGIIIb light-green), and previously reported isolates VGIIIa reference strain B4546 (Black) and VGII (Grey). (C) Ranked IPR of tested *C. gattii* isolates.

Virulence attributes of clinical and environmental *C. gattii* isolates were ascertained based on intracellular proliferation rates (IPR) and percent mitochondrial tubularization within J774 BALB/c macrophage cell lines. An IPR≥2 indicates a high replication rate within macrophages and is frequently correlated with higher virulence. An IPR score <1 indicates low proliferation levels were observed in macrophages, which have been correlated with a lower potential for virulence in whole animal models in previous studies. An IPR between 1 and 2 indicates moderate proliferation in macrophages and moderate virulence attributes. We observed moderate to low IPR for selected VGIIIb and VGIIIa isolates in direct comparison to the highly virulent VGIIa *C. gattii* major Pacific Northwest outbreak isolate R265 (Figure 7a and 7b). Percent yeast cells observed with tubularized mitochondria ranged from 9 to 37%, which suggests mitochondrial tubularization is decoupled from IPR in macrophages for *C. gattii* VGIII isolates compared to the VGIIa isolate R265 [21], [34] (Figure 7b). The coefficient of determination (R^2^ = 0.2593 is <1, p = 0.0155) indicates a weak positive linear correlation exists between IPR and the presence of tubular mitochondria in all VGIII isolates. A positive but weakly linear correlation is observed for VGIIIa (R^2^ = 0.3692 is <1, p = 0.0624) but not for VGIIIb (R^2^ = 0.001 is <1, p = 0.9226) suggesting differences in IPR and mitochondrial regulation may be associated with different molecular sub-types. Our results demonstrate that survival and replication in macrophages (IPR) is similar between environmental and clinical VGIIIa and VGIIIb isolates of both mating types. Notably some VGIII strains have high rates of tubularization prior to a known encounter with the macrophage niche (environmental isolates) while others do not display increased tubularization after encountering the macrophage niche (clinical isolates). The presence of tubular mitochondria did not appear to be strongly correlated with IPR in VGIII isolates, in contrast with previous studies on VGII Pacific northwest outbreak isolates [21], [34]. These data may reflect that VGIII isolates commonly infect HIV/AIDS patients versus VGII isolates that rarely occur in HIV/AIDS patients, and hence VGIII isolates may be in general less virulent than VGII isolates.

### *C. gattii* VGIII isolates are virulent in the murine model

The virulence attributes of VGIII *C. gattii* isolates were further addressed in vivo utilizing the murine intranasal model. The first two environmental isolates, MCP-1A and 78-1-S3C (both identified in late 2011), were assessed for virulence in the BALB/c mouse model and the average length of survival ranged from 56 to more than 250 days post infection (Figure S6a and S6b). Mean survival ranked from shortest to longest is as follows: CA1232 (84 days), CA1053 (90.5 days), CA1308 (96 days), CA1508 (136 days), BHPP1-S2B (149 days), NIH191 (173.5 days), BHPP3-S1A (197 days) and all others were undetermined because some mice were still surviving at the termination of the experiment. MCP-1A and 78-1-S3A were less virulent than the VGIII clinical isolates RR and VV (p<0.002, Figure S6b). Environmental isolate 78-1-S3A was similar in virulence to clinical isolate CA1089 (p = 0.6409) and somewhat more virulent than the historical NIH312 isolate (p = 0.034) (Figure S6b). Our results indicate that environmental isolate MCP-1A is less virulent than environmental isolate 78-1-S3A (p = 0.0083) with which it shares an indistinguishable MLST profile, IPR, and differs by less than 90 SNPs at the whole genome level (Figure 5, Table S8), suggesting that relatively few genetic differences could be responsible for differences in virulence between two very closely related isolates. At a minimum, no more than 46 SNPs seemed to be necessary to confer a phenotypic difference (Figure 5, Table S7). Additionally, differences in epigenetic or transcriptional modifications could contribute to phenotypic differences observed between matched MLST isolates. *C. gattii* VGIII environmental isolates exhibited attenuated virulence in comparison to VGIII clinical isolates in the intranasal murine mouse model. Virulence observed in the mouse model correlated with previously determined moderate IPR and was decoupled from mitochondrial phenotype suggesting roles of intracellular proliferation and mitochondrial program in the divergence in virulence of *C. gattii* molecular types.

**Figure 8:**
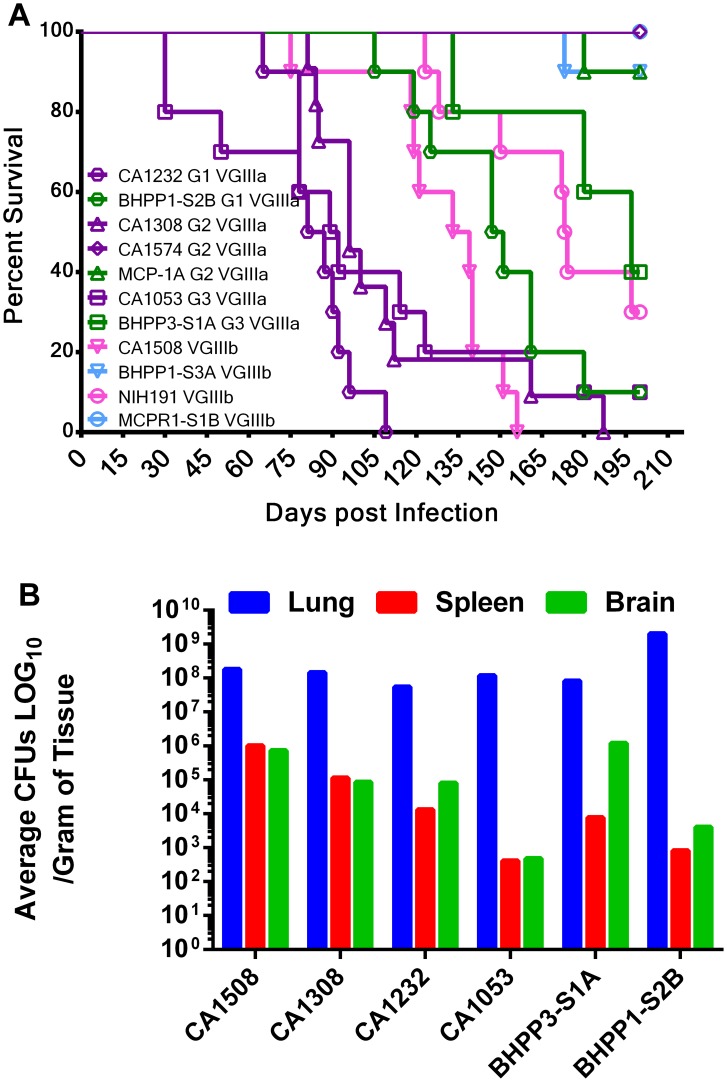
Environmental isolates that share an indistinguishable MLST profile with clinical isolates are less virulent in mice. (A) 11 matched isolates representing four distinct MLST groups, three VGIIIa (G1, G2, G3, two strains each) and one VGIIIb (4 strains, 2 clinical, and 2 environmental), were tested for virulence in the murine animal model. Ten male A/JCr mice per strain were intranasally infected with 10^6^ cells and survival was recorded for 200 days. MLST groups are represented by shared symbol and group number. Clinical isolates (VGIIIa dark-purple; VGIIIb light purple), environmental isolates (VGIIIa dark-green; VGIIIb light green). Median survival CA1232 = 90 days, CA1308 = 100 days, CA1053 = 103 days, CA1508 = 127 days, and BHPP1-S2B = 138 days. (B) Mice were assessed for tissue burden postmortem.

Virulence attributes of closely related environmental versus clinical genotypes were further examined in A/JCr mice, which are typically more permissive to cryptococcal infection than BALB/c mice. Four groups of MLST matched strains representing VGIIIa (group 2, CA1308, CA1574, and MCP-1A; group 3, CA1053 and BHPP3-S1A) and VGIIIb (group 1, CA1232 and BHPP1-S3A; group 4, CA1508, MCPR1-S1B, NIH191/ATCC32608) were compared in the A/JCr murine model (Figure 8a). Clinical isolates exhibited reduced survival time and higher virulence in comparison to MLST matched environmental isolates. More virulent clinical isolates were associated with more virulent environmental isolates (Figure 8a). MCP-1A and BHPP1-S3A were severely attenuated for virulence in comparison to other virulent strains with 9 of 10 infected animals surviving to the conclusion of the study. Two isolates, one environmental (MCPR1-S1B) and one clinical (CA1574), were avirulent for the duration of study (250+ days post infection) in A/JCr mice (all 10 of 10 animals infected surviving). Tissue burdens ranged from 10^7^ to 10^8^ cells/gram tissue in the lungs, 10^2^ to 10^6^ cells/gram tissue in the spleen, and 10^2^ to 10^6^ cells/gram tissue in the brain and varied greatly between isolates. Consistently, and in agreement with previously published studies, higher organ loads were observed in the lung tissue in comparison to the brain tissues and may suggest that pulmonary cryptococcosis could contribute to the observed mortality [22], [68]. Granulomas were observed macroscopically in the lungs post necropsy and microscopically in histopathological sections for both VGIIIa and VGIIIb isolates (data not shown). Although we observed differences in mean survival time between VGIII strains, high tissue burdens were observed for all strains (of both high and low virulence) post mortem (Figure 8b). This suggests that determination of virulence is not just a static predetermined trait but also dependent on duration of exposure and host status, which could play a more prominent role in disease development in longer-lived human and animal populations.

### Response of *C. gattii* VGIIIa and VGIIIb molecular types to antifungal drugs

**Figure 9:**
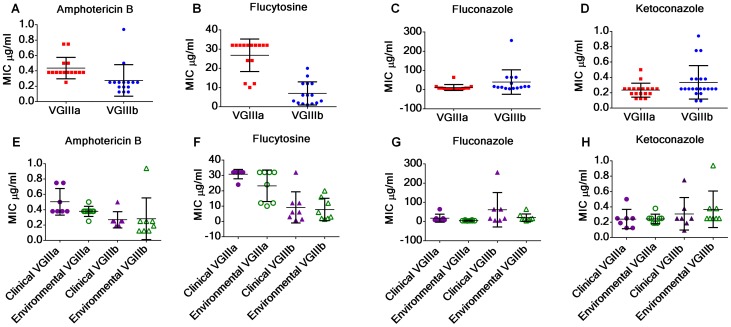
*C. gattii* VGIIIb isolates display higher antifungal susceptibility values to Amphotericin B and flucytosine in contrast to VGIIIa isolates. (A, E) MIC of VGIIIa (red) versus VGIIIb (blue) for amphotericin B (A), flucytosine (B), fluconazole (C), and ketoconazole (D). MIC of clinical (purple) and environmental (green) isolates of VGIIIa (triangles) and VGIIIb (circles). The MIC of VGIIIa isolates was significantly higher than VGIIIb isolates for amphotericin B (p = 0.0178) and flucytosine (p<0.0001) but not for fluconazole (p = 0.1059) or ketoconazole (p = 0.0685). No significant differences were observed between environmental and clinical isolates of VGIIIa or of VGIIIb in response to amphotericin B (E), flucytosine (F), fluconazole (G), or ketoconazole (H) (Table S10a, S10b and S10c). MIC values, which exceeded the maximum concentration for each Etest, were assigned as the maximum concentration tested for averaging and graphing (Table S1). Maximum concentrations of Etest amphotericin B (32 µg/ml), fluconazole (256 µg/ml), flucytosine (32 µg/ml), and ketoconazole (32 µg/ml).

*Cryptococcus* is an environmentally acquired fungal pathogen of humans and infections caused by differing molecular types may result in differences in clinical presentations, treatment, and therapeutic outcomes. In this study, the antifungal susceptibilities to amphotericin B, fluconazole, flucytosine, and ketoconazole were assessed for *C. gattii* VGIII isolates to examine if differences in antifungal susceptibility might be associated with molecular type and/or origin of the isolates (environmental or clinical). Our results provide evidence that the MIC of VGIIIa isolates can be higher than VGIIIb for recommended drug therapies, amphotericin B (p = 0.0178, Figure 9a) and flucytosine (p<0.0001, Figure 9b), but not for the maintenance antifungal drug fluconazole (p = 0.1059, Figure 9c), or the second line drug ketoconazole (p = 0.0685, Figure 9d). No significant differences were observed between clinical and environmental isolates within VGIIIa or VGIIIb isolates suggesting the genetic propensity for antifungal resistance may exist in environmental populations (Figure 9e–h, p>0.05). However, resistance was observed in response to exposure to fluconazole and/or flucytosine in VGIIIb clinical (HM-1, 7180567, 7618666, CA1508) and environmental isolates (MCPR1-S1B, MCPR1-S2B, MCPR1-S2A, BHPP1-S2A, BHPP1-S3A) as well as VGIIIa clinical (KB-1, CA1308) and environmental (MCP-1A, BHPP1-S2B, BHPP3-S2A) isolates suggesting some isolates may have a greater propensity for the microevolution of drug resistance within the host, possibly influencing long term treatment outcomes. In summary, we observed the emergence of resistance (fluconazole and flucytosine) and higher MICs (amphotericin B and flucytosine) among different subsets of the *C. gattii* VGIII population for the recommended first line treatment drugs, amphotericin B and flucytosine, suggesting molecular type and the genetic propensity for resistance could possibly impact treatment and long-term outcomes.

## Discussion

The clinical and veterinary prevalence of cryptococcosis and the identification of *C. gattii* as the causative agent in the PNW outbreak stimulated increased environmental sampling that resulted in the identification of the ecological niche of *C. gattii* in the environment in association with native trees on Vancouver Island and in the Pacific Northwest [59]. Increased molecular typing has resulted in the identification of both *C. gattii* VGIIa and VGIIb molecular types with shared MLST profiles between clinical and environmental isolates, indicating the endogenous environmental reservoir for the ongoing *C. gattii* PNW outbreak [22], [26], [60]. The outbreak is caused by three largely clonal lineages: VGIIa/major, VGIIb/minor, and VGIIc/novel which are members of a global, fertile, recombining population that gave rise to the hypervirulent outbreak strains [21].

Previous studies have demonstrated the presence and molecular diversity of *C. gattii* in the clinical population of Southern California but only one historical environmental VGIII isolate (WM161) had been reported from this region, even with increased sampling [22], [45], [69]–[71]. The historical VGIII environmental isolate (WM161) differed in MLST profile (due to unique *IGS* and *GPD1* alleles) compared to all known VGIII clinical isolates from California. Ongoing epidemic incidences of *C. gattii* that have emerged in the Western USA are cause for concern due to clinical infections in both immunocompetent individuals and populations with immunocompromised status, including HIV/AIDS. *C. gattii* is an environmentally acquired pathogen and thus the paucity of environmental isolates is incongruent with its known presence in the clinical population in California. The identification and environmental isolation reported herein of *C. gattii* VGI and VGIII from four independent sampling sites in the greater Los Angeles area mimics the patchy and often sporadic isolation reported for other environmentally acquired fungal pathogens (*Blastomyces*, *Coccidioides*) associated with periodic outbreaks in the USA [6], [72]–[74].

In this study we report the environmental isolation of 19 *C. gattii* VGIIIa, VGIIIb, and VGI isolates from Los Angeles, California from samples obtained from three novel tree species *Pinus canariensis* (Canary Island pine), *Liquidamar styraciflua* (American sweetgum), and *Metrosideros excels* (Pohutukawa tree). These environmental isolates were obtained from areas in close proximity to the residences of patients. We have identified an environmental source of the most common *C. gattii* VGI molecular type, which constitutes a smaller proportion of *C. gattii* infections in California and elsewhere in the US [22], [45], [75], [76]. Of the 19 environmental isolates, 16 share indistinguishable MLST profiles with previously reported clinical isolates from the Southern California region identifying endogenous environmental reservoirs for both VGIIIa and VGIIIb infections in the Los Angeles, metropolitan area, USA [22]. Therefore, we have the rare opportunity to identify and genetically link known environmental reservoirs to individual cases in Southern California.

In this study we report the presence of fertile *MAT* **a** and *MAT*α environmental isolates and demonstrate evidence of recombination within the VGIIIb population in the Southern California region. This is notable, at least in part, because **a**-α mating is relatively rare in the *Cryptococcus* species complex, with a few notable exceptions, because of the paucity of *MAT* **a** isolates [56], [58], [77], [78]. In the case of *C. neoformans* var. *grubii*, the majority of *MAT* **a** isolates are part of a specific population restricted to Botswana that is also hypothesized to be the origin of the species [79]. The population of VGIIIb *MAT* **a** isolates could similarly represent a locally restricted founder population in California. It is also possible that the paucity of *MAT* **a** isolates within the VGIIIa population, may be indicative of a high frequency of α-α mating in the population. As in VGIIIb, there is evidence for recombination within the VGIIIa population, but in this case may be occurring without the involvement of *MAT* **a** isolates. While we cannot rule out the existence of *MAT* **a** isolates that have remained unsampled, our current evidence suggests this population may reassort without bisexual mating. Further sampling will increase the power of this analysis Sex within *C. gattii* populations is important for two reasons. First, the sexual cycle produces spores that can be aerosolized as potential infectious propagules [80]. Second, sex can aid in the evolution of virulence. Same-sex mating has been proposed as a factor in the development of the VGIIa major outbreak strain in the Pacific Northwest, while **a**-α sexual reproduction aids in the transmission of hypervirulence [25], [33]. Through both routes, sex can contribute to the development and persistence of an outbreak. In addition, sexual reproduction led to the emergence of the modern pathogenic lineage for the common human parasite *Toxoplasma gondii* and selfing has recently been shown to drive outbreaks [81], [82].

*C. gattii* VGIII clinical and environmental isolates share similar in vitro IPR and mitochondrial phenotypes. Moderate ranges of IPR appear unrelated to the extent of mitochondrial tubularization in contrast to *C. gattii* VGII isolates, suggesting these VGIII isolates have reduced virulence in comparison to the VGII outbreak strains [21], [33]. We observed reduced virulence of VGIII in comparison to VGII in the murine model, consistent with the observed predilection of VGIII to infect immunocompromised individuals with HIV/AIDS compared to VGII that commonly infects otherwise healthy patients. An alternative explanation is that *C. gattii* VGIII causes dormant infections that can be reactivated at the time of immunosuppression whereas VGII causes primary infections. However, this alternative explanation seems less likely because VGII has also been associated with the potential to cause dormant infections [14]. We tested paired clinical and environmental isolates that shared indistinguishable MLST profiles, and although WGS indicated relatively few genetic differences, all clinical isolates were more virulent in comparison to the environmentally isolated paired MLST strains except for VGIIIa CA1574. Furthermore, in the paired MLST matched isolates that were analyzed in murine virulence assays we found that all VGIIIa strains were more virulent than VGIIIb strains, in agreement with previous findings [22]. Tissue burden and histopathological analyses indicated significantly higher fungal burdens in the lungs in comparison to the spleen or brain, consistent with previous reports of a predilection of VGIII to initiate prolonged pulmonary infections [22], [32]. Thus, although clinical and environmental isolates shared indistinguishable MLST profiles, IPR, and >99.9% sequence identity, their virulence potential differed in the murine model.

Whole genome sequencing of three different MLST matched groups representing both VGIIIa and VGIIIb populations indicated that previously reported clinical isolates (circa 2000–2005) and newly identified environmental isolates (2011–2012) are extremely similar, differing at only 46 SNPs between VGIIIa clinical isolate CA1053 and environmental isolate BHPP3-S1A, and 183 SNPs between the VGIIIb clinical isolate CA1508 and the environmental isolate MCPR1-S1B. This is all the more poignant given that the clinical and environmental isolates were ascertained over an isolation period spanning 5 to 12 years, further forging a link between the environmental isolates and clinical infections in Southern California. This can be compared to H99 lab passaged isolates obtained over a ten year period, which are distinguished by 11 SNPs and 11 indels [83]. Furthermore, a recent report on *Staphylococcus aureus* (genome size 2.4 Mb) used a cutoff of 40 single nucleotide variants (SNVs) to decide whether two strains were indistinguishable or different [84]. We aligned our sequences to R265 with a total genome size of 17.5 Mb, which would allow for 6.2-fold more variants and up to 249 SNVs. Thus, by even this strict comparison the VGIIIa/b clinical and environmental isolates are closely related than bacterial isolates concluded to be causally related in chains of transmission.

Whole genome sequencing allows new approaches to pathogenesis. However, comparative genomic approaches have limitations. When comparing highly pathogenic and less pathogenic isolates, finding a genetic change important for pathogenesis is challenging because of unrelated incidental genetic diversity. One method to avoid this is use strains that have a common, recent origin. This reduces genomic differences that could obscure causative genotypic changes and increases the probability of linking genotype to phenotype. In this study, environmental isolates that we propose directly gave rise to the infectious isolate were chosen via MLST matching; however, similar approaches could be used for longitudinal studies of individuals with recurrent fungal infections or for other environmental infections where a source population is readily available. While beyond the scope of this study, resource populations established through MLST matching of isolates with disparate phenotypes will provide an avenue for linkage between genotype and phenotype. Substantial differences in virulence were observed in our studies between two very similar isolates. At a minimum, no more than 46 SNPs seems necessary to confer a phenotypic difference between the clinical isolate CA1053 and the environmental isolate BHPP3-S1A; only 15 of these SNPs result in nonsynonymous changes. We sequenced three closely related VGIIIa isolates from group 2, representing two independent environmental isolates obtained from different locations and a previously identified clinical isolate. The three isolates differ by only 90 SNPs but exhibit differences in pathogenicity and antifungal sensitivity. Furthermore this genotype is noteworthy because it is represented in the current clinical population by isolate 7489719. This suggests that adaptation from an environmental lifestyle to a pathogenic lifestyle may be a very rapid event, involving epigenetic modification, changes in transcriptional regulation, or requiring few genetic changes. Passage of *Cryptococcus* through amoeba, nematodes, slime molds, plants, and animals yields passaged strains with enhanced virulence [68], [85]–[89]. Prolonged lab culture without host exposure can attenuate virulence [90]. Passage of strain H99 resulted in a lineage with enhanced or attenuated virulence with only 4 associated SNPs, two of which could contribute to attenuated virulence [83]. Also notable is that none of the SNPs from our three matched sets was shared between sets or in common genes. This suggests that either there are multiple independent routes of adaptation to produce a more virulent organism, or that transcriptional and epigenetic changes may also contribute.

*C. gattii* is an environmentally acquired opportunistic fungal pathogen that can cause acute or latent infections in both immunocompromised and immunocompetent hosts and increased recognition of ongoing outbreaks in humans, pets, and wildlife is emerging as a serious public health concern and financial burden. We report the isolation of fertile *C. gattii* VGIIIa, and VGIIIb isolates from environmental soil and swab samples from non-*Eucalyptus* host trees in the greater Los Angeles area of Southern California. MLST and WGS analysis coupled with mating and virulence studies demonstrate that environmental *C. gattii* VGIII isolates are actively recombining and the likely source of human acquired infections in this region and can further contribute to ongoing infections in Southern California. There is now a substantial body of accumulated data that suggest that *C. gattii* is associated in the environment with many tree species (native and non-native) worldwide and these hosts function as environmental reservoirs for disease outbreaks. Future environmental and epidemiological studies can further incorporate molecular and whole genome analysis to connect known clinical/veterinary incidences of cryptococcosis with newly identified ecological niches to further address the link between genotype, risk of exposure, and propensity for initiating disease.

## Materials and Methods

### Environmental sampling and isolates utilized in this study

Swabs of individual tree trunks and soil samples from around the base of trees were collected during the summers of 2011 and 2012 utilizing BD BBL Single application CultureSwabs with Liquid Amies (VWR#90001-036). In summary, over two years 24 sites were sampled to obtain 109 tree swabs of over 30 tree species and 58 soil samples from collected from the greater Los Angeles area. In 2011 samples were obtained from 9 locations, 64 trees (30 different species, including 10 *Eucalyptus* trees from four independent sites.), and 25 soil samples in the greater Los Angeles area. In 2012 the two sites that had previously yielded *C. gattii* were resampled and 15 additional sites were sampled focusing on *Pinus canariensis* and *Liquidambar styraciflua*. From these trees 45 trees were swabbed and 33 soil samples were collected. The swabs were streaked onto Niger seed (NGS) agar containing chloramphenicol (0.5 g/L, Sigma C0378-100G) Two grams of soil were suspended in 10 mL of sterile ddH_2_0 and allowed to settle. Three 100 µl aliquots were plated on NGS agar. Plates were incubated at 30°C for 1 to 3 days. Yeast colonies producing brown pigmentation were selected and colony purified. All clinical and environmental isolates were streaked onto NGS and canavanine-glycine bromothymol blue (CGB) agar and incubated for 1 to 3 days to identify *C. gattii* isolates. Clinical isolates were obtained from 48 patients who were treated at the University of Southern California, Harbor-UCLA Medical Center, or Kaiser Permanente Downey Hospital between February 2008 and January 2013. Of these patients, 32 were considered to be immunocompromised due to HIV/AIDS or other causes. The clinical isolates were de-identified and linked to major cross streets near the patients' homes. Stocks were maintained at −80°C in 25% glycerol. All strains utilized in this study are listed in Table S1. Genomic DNA was prepared by the CTAB method for all isolates. Potential *C. gattii* isolates were screened for molecular type by size differences of the *ATP6* PCR product [33], [61].

### Multilocus sequence typing analysis

Multilocus sequence typing was performed on 12 loci (*SXI1*α or *SXI*2**a**, *IGS*, *TEF1*, *GPD1*, *LAC1*, *CAP10*, *PLB1*, *MPD1*, *CAP59*, *TOR1*, *SOD1*, and *URA5*) [15], [21], [25]. For each isolate, genomic regions were PCR amplified, purified (ExoSAP-IT, Qiagen), sequenced, and both forward and reverse strands were assembled with complete double-strand coverage. All primers utilized in this study are listed in Table S2. MLST sequences were viewed and edited by Sequencher; alignment and phylogenetic analyses were conducted using MEGA version 5 [91]. Each allele was assigned a corresponding number, or given a new number if the sequence was not already assigned an allele number in the GenBank database (NCBI), International Society for Human and Animal Mycoses MLST Database (ISHAM), or previously published reports [15], [22], [27], [45]. GenBank accession numbers with corresponding allele numbers are listed in Table S3. Haplotype network analysis was performed using TCS software (version 1.21) [92].

### Ploidy analysis

Ploidy was determined by fluorescence activated cell sorting (FACS) as previously described [93]. Briefly, cells were grown, passaged twice in 25 ml YPD broth at 25°C, collected by centrifugation, and washed and resuspended in 5 mL of 1x PBS. 1 mL of cells was collected by centrifugation and fixed in 70% ETOH overnight with gentle shaking at 4°C. Cells were collected by centrifugation, washed, and resuspended in 1 mL of 1x NS buffer. Cells were centrifuged and resuspended 200 µl (180 µl NS buffer, 14 µl RNase A, 6 µl propidium iodide and incubated overnight at room temperature. 50 µl of each strain (10,000 cells) was mixed with 500 µL of Tris-PI mix (482 µl 1M Tris pH 7.5+18 µl propidium iodide) and analyzed using the FL1 channel (slow laser scan) on a Becton-Dickinson FACScan. *C. gattii* NIH444 and *C. neoformans* XL143 were utilized as haploid and diploid reference controls.

### Sequencing and assembly of VGIII genomes

Whole Genome Sequencing (WGS) was done by Illumina paired end reads. Genomes of strains CA1053, CA1308, CA1508, BHPP3-S1A, MCP-1A, 78-1-S3A, MCPR1-S1B, and 97/433 were sequenced using HiSeq2500 using paired end reads with an insert size of approximately 300 bp and a read length of 100 bp. Initial processing was performed using the Illumina Pipeline (v.1.8.2) [94]. The genome of B4546 was previously sequenced [33]. Reads were aligned to the published VGII reference genome supercontigs using BWA-sampe [95] and SNPs were called using the Genome Analysis Toolkit (GATK V2.4-9) Unified Genotyper with the haploid ploidy setting. The resulting calls were then filtered to remove calls with a quality score below 30, and individual depths below 5 reads. SNPs common to each pair were removed and the remaining SNPs differentiating the subset were manually examined using the BAM files to remove erroneous calls based on poor mapping or repetitive sequences. The resulting variants were analyzed using SnpEff to determine impact of SNPs [96]. Assignment of gene function was carried out using a combination of BLAST to the *S. cerevisiae* genome and CD search through NCBI.

Whole genome trees were generated from a 100 kb region aligning to supercontig 5 of the VGII reference genome. SNPs were manually filtered to eliminate erroneous calls. The resulting SNPs were used to generate alternate reference sequences using GATK's FastaAlternateReferenceMaker that were then aligned using Kalign [97]. The resulting alignment was used to produce a maximum likelihood tree using MEGA5 with 500 bootstraps [91].

### Allele compatibility tests

Allele information derived from MLST analysis was used to construct paired allele networks, where alleles present in combination were connected with a solid line. The presence of all four combinations of two alleles was represented as an hourglass shape, with the lines darkened to indicate evidence for recombination [22]. This analysis relies on the assumption that homoplasy is a rare event.

### Mating assays

*MAT* **a** and *MAT*α isolates were grown on YPD agar at room temperature for two days. Cells were scraped off YPD agar, washed, and suspended in autoclaved ddH_2_O. Cells were spotted alone or in combination (*MAT*α and *MAT* **a**) on V8 pH = 5 and V8 pH = 7 mating agar and incubated in the dark at room temperature. Plates were checked once a week for 24 weeks for hyphae and/or the formation of basidia and basidiospores. To examine mating reactions for morphological features associated with mating, scanning electron microscopy studies were conducted. One centimeter square blocks were excised from the agar plates and fixed in 2% glutaraldehyde (Electron Microscopy Sciences, EMS, Hatfield, PA, USA) with 0.05% malachite green oxalate (EMS) in 0.1 M sodium cacodylate buffer and incubated at 4°C until further processing. The fixation buffer was removed, blocks were dehydrated by ethanol series, critical point dried (Pelco CPD2, Ted Pella, Inc., Redding, California, USA), sputter coated, and imaged with the FEI XL30 SEM-FEG (FEI Company, Hillsboro, Oregon, USA) at the electron microscopy facility at North Carolina State University.

### *In vitro* macrophage intracellular proliferation rate and formation of tubular mitochondria

Intracellular proliferation rate (IPR) was determined as previously described utilizing J774 macrophages [21], [33], [34]. Macrophages were co-incubated for 2 hours with opsonized cryptococcal cells (18B7 antibody) as described previously [21], [34]. Wells were washed with phosphate-buffered saline (PBS) four times to remove excess extracellular yeast cells and 1 ml of fresh serum-free DMEM was then added. For the control time point T = 0, the DMEM was discarded and 200 µl of sterile ddH_2_O was added to lyse the macrophages. After 30 minutes, lysed macrophages released intracellular yeast cells, which were then collected in an additional 200 µl ddH_2_O. For the additional time points T = 18 hrs and 24 hrs, intracellular cryptococcal cells were collected and independently counted with a hemocytometer.

The IPR assay was replicated at least three times for each strain tested using independently propagated batches of macrophages. The IPR value was calculated by dividing the maximum intracellular yeast number by the initial intracellular yeast number at T = 0. We confirmed that Trypan Blue stains 100% of the cryptococcal cells in a heat-killed culture, but only approximately 5% of cells from a standard overnight culture. Compared to a conventional colony counting method, this method was shown to be more sensitive in detecting the clustered yeast population or yeast cells undergoing budding.

Tubularized mitochondria were determined as previously described [33], [34]. *C. gattii* cells were grown overnight at 37°C in DMEM, harvested, washed twice in PBS, and re-suspended in PBS plus Mito-Tracker Red CMXRos (Invitrogen) at a final concentration of 20 nM for 15 min at 37°C. Cells were washed three times and re-suspended in PBS. 100 yeast cells in three replicates per strain were randomly chosen, imaged using a Zeiss Axiovert 135 TV microscope with a 100X oil immersion Plan-Neofluor objective or a Nikon Eclipse T*i* Plan Apo VC 60X oil immersion objective, images were collected, and percent tubularized mitochondria were calculated. All images were processed identically in ImageJ and mitochondrial morphologies were analyzed and counted blindly. IPR and tubularization data were analyzed for statistically significant differences using one-way ANOVA analysis with multiple comparisons by Tukey's Honestly Significant Difference (HSD) posthoc test. A p-value of <0.05 after controlling for multiplicity was considered to be statistically significant. IPR and percent yeast cells with tubular mitochondria were plotted and linear regression, residual plot, and R^2^ analysis was completed with GraphPad Prism version 6.03 (Windows, GraphPad Software, La Jolla California USA, www.graphpad.com.).

### *In vivo* murine model

Six-week-old female A/JCr mice (Cat. No. 01A24, NCI-Frederick) or male BALB/c mice (Cat. No. 01B05, NCI-Frederick) were used. Mice were acclimatized in the facility for one week prior to infection by intranasal instillation and were housed in cages at 21°C and 50% humidity with a 12 hr light/12 hr dark cycle. Cells were grown in YPD broth with two successive passages and collected by centrifugation, washed with autoclaved ddH_2_O, and suspended in autoclaved ddH_2_O. Mice were inoculated intranasally with 10^6^ cells in 40 µl. At the first signs of poor health or discomfort, mice were euthanized with CO_2_. Kaplan-Meier survival curves were constructed by GraphPad Prism version 6.03 (Windows, GraphPad Software, La Jolla California USA, www.graphpad.com.). Additional data on colonization was obtained from lung, brain, and spleen tissues postmortem. Tissues were aseptically harvested postmortem. Tissue homogenates were serially diluted and plated on YPD agar, incubated at 30°C for 2 to 3 days, and colony forming units (CFUs) were determined. Two mice were chosen at random from each treatment group, and lung, spleen, or brain tissues were fixed in 10% buffered formalin (lung and spleen tissues) or Bouin's fixative (brain tissues), processed into paraffin blocks, sectioned, and stained with hematoxylin and eosin (H & E) and Mayer's mucicarmine for histopathological examination.

### Ethics statement

All animal studies were conducted in the Division of Laboratory Animal Resources (DLAR) facilities at Duke University Medical Center (DUMC) and animals were handled according to the guidelines defined by the United States Animal Welfare Act and in full compliance with the DUMC Institutional Animal Care Use Committee (IACUC). Animal models were reviewed and approved by DUMC IACUC under IACUC protocol # A217-11-08.

### Antifungal drug sensitivity

*C. gattii* isolates were grown and passaged two times in YPD broth at 30°C. Cells were collected by centrifugation and washed and suspended in 0.85% NaCl to an OD_600_ = 2 (equivalent to 1 McFarland turbidity) following BioMerieux recommended protocol (http://www.biomerieux-diagnostics.com). Two RPMI agar plates (RPMI 1640+MOPS+2% Glucose+1.5% agar) per strain were swabbed in three contrasting directions and allowed to dry at room temperature. Amphotericin B, fluconazole, flucytosine, and ketoconazole BioMerieux Etest strips were place on plates in pairs, and incubated 48–72 hours at 35°C with 5% CO_2_ and MIC values were reported as indicated in the Etest instructions.

## Supporting Information

Figure S1 **Geographic sampling area.** (A) Swab and soil samples collected from 24 locations throughout the greater Los Angeles area, California, USA (Black circle).109 trees and 58 soil samples were obtained. Map data NationalAtlas.gov. (B–F) *C. gattii* environmental isolates were associated with non-*Eucalyptus* hosts. (B, C) 78-1-S3A was isolated from swab samples of *Liquidambar styraciflua* (American sweet gum) and MCP-1A (D, E, F) was isolated from soil samples of *Pinus canariensis* (Canary Island pine). Locations of positive environmental samples and residences of known clinical infections (G). Locations of known clinical cases noted with triangles and environmental isolates with circles. Different colored markers indicate molecular types associated with the particular location Blue = VGIIIb, Red VGIIIa, and green = VGIIIc. Map data NationalAtlas.gov.
(PDF)

Figure S2 **Phylogenetic and haplotype network analysis of** ***ATP6*** **sequences.** (A) Phylogenetic tree for *ATP6* using the Maximum Likelihood (MLC) method based on Tamura-Nei model applying Neighbor-Join and 500 bootstrap replicates. The 500 bootstrap tree with the highest log likelihood is shown. Analysis involved 53 nucleotide sequences and 612 positions. Scale bar indicated 0.005 substitutions per nucleotide. Mitochondrial Haplotype (HP) allele number consistent with Bovers et al. 2009. (B) Haplotype network analysis of *ATP6* sequences. Alleles are colored red representing VGIIIa, blue representing VGIIIb, and dual-coloration representing alleles shared between the VGIIIa and VGIIIb molecular types. Alleles in squares represent the proposed ancestral allele, circles represent alleles present in the population, lines between alleles represent one predicted evolutionary event, and smaller black circles represent alleles that have not been recovered, or which may no longer be represented in the population.
(TIF)

Figure S3 **Additional paired allele diagrams.** Informative paired allele diagrams depicting MLST data. An hourglass shape indicates that all four allele combinations were observed (AB, ab, Ab, aB), providing evidence for recombination. Alleles unique to California are circled, while black lines indicate combinations found both in Californian isolates and those from elsewhere. Green lines indicate combinations found only in California, and orange lines indicate combinations only found outside of California.
(TIF)

Figure S4 **Additional haplotype networks of MLST alleles not depicted in** **Figure 6** **depict that majority of VGIIIa and VGIIIb alleles are not shared and segregate according to molecular type.** Alleles for each respective locus are indicated numerically. Alleles are colored red representing VGIIIa, blue representing VGIIIb, and dual-coloration representing alleles shared between VGIIIa and VGIIIb molecular types. Squared alleles represent the proposed ancestral allele, circles represent alleles present in the population, lines between alleles represent one predicted evolutionary event, and smaller black circles represent alleles that have not been recovered, or which may no longer be represented in the population. Haplotype analysis of the majority of MLST loci (*LAC1*, *SXI1*, *CAP59*, *SOD1*, *MPD1* and *IGS1*) designate VGIIIb alleles as ancestral in comparison to *URA5*, *TOR1*, and *GPD1* for which the designated ancestral allele belongs to the VGIIIa population.
(TIFF)

Figure S5 **Additional evidence that environmental** ***C. gattii*** **isolates are fertile.** Scanning electron micrographs depict microscopic characteristics of mating. Fused clamp connections are observed in matings between 78-1-S3A×B4546**a** (A) and 78-1-S3A×JEC20**a** (B). Basidia and basidiospores are observed for 78-1-S3A×JEC20**a** (C) and MCP-1a×JEC20**a** (D) and 78-1-S3A×RR (E). In addition, to basidia and basidiospores observed in Figure 6, matings between MCP-1A×B4546a (F) also produce bald basidia (without spores) and unfused clamp connections in some sections of the mating colony.
(TIFF)

Figure S6 ***C. gattii*** **environmental isolates are less virulent in BALB/c mice in comparison to clinical isolates.** (A) Average survival time of two Californian environmental isolates in BALB/c mice in comparison to previously reported strains NIH312, RR, and VV. Clinical isolates (VGIIIa dark-purple; VGIIIb light-purple), environmental isolates (VGIIIa dark-green), and VGIIIb reference strain NIH312 (black). Average survival +/− SEM is plotted. (B)The Kaplan-Meier survival curves are presented. Ten male BALB/c mice per strain were intranasally infected with 10^6^ cells and survival was recorded for 252 days. At the termination of the experiment some mice were still surviving isolate CA1089 (n = 2), NIH312 (n = 6), or 78-1-S3A (n = 2).
(TIFF)

Table S1 **Strains utilized in this report.**
(TIF)

Table S2 **Primers utilized in this study.**
(TIFF)

Table S3 **List of GenBank accession numbers and allele designations for all isolates sequenced in this report.**
(TIF)

Table S4 **Extended MLST table including ploidy and** ***ATP6*** **PCR product length of newly identified** ***C. gattii*** **strains.** Multilocus sequence typing was performed on 4 additional MLST loci (*CAP59*, *TOR1*, *SOD1*, and *URA5*) to extend analysis to a total of 12 MLST loci. Unique alleles were assigned a distinct color for each marker. FACS was performed on all isolates utilizing standard haploid (EJB18) and diploid (XL143) controls. *ATP6* PCR was amplified utilizing Primers ATP6-F and ATP6-R as described in Table S2.
(TIF)

Table S5 **Global molecular analysis of** ***C. gattii*** **VGI isolates endemic to Southern California.** Multilocus sequence typing was performed on 8 loci. Unique alleles were assigned a distinct color for each marker. New environmental (green, n = 3) VGI isolates from California are compared to previously analyzed VGI isolates.
(TIFF)

Table S6 **SNPs observed from whole genome sequencing of MCPR1-S1A and CA1508.** Whole genome sequencing of MLST matched VGIIIa group 1 pairs of environmental and clinical isolates.
(TIFF)

Table S7 **SNPs observed from whole genome sequencing of BHPP3-S1A and CA1053.** Whole genome sequencing of MLST matched VGIIIa group 2 pairs of environmental and clinical isolates.
(TIFF)

Table S8 **SNPs observed from whole genome sequencing of 78-1-S3A, MCP-1A, and CA1308.** Whole genome sequencing of MLST matched trio of VGIIIb environmental and clinical isolates.
(TIFF)

Table S9 **Table of productive/fertile crosses.** Many VGIII isolates appear fertile when crossed with known tester strains.
(TIFF)

Table S10 **Antifungal sensitivity of** ***C. gattii*** **isolates.** (A) VGIIIa and VGIIIb MIC µg/ml results for *C. gattii* isolates as determined by Etest. (B) Average MIC values of VGIIIa, VGIIIb, clinical and environmental isolates. (C) P values calculated by GraphPad Prism version 6.03 for each group. Maximum concentrations of Etest were amphotericin B (32 µg/ml), fluconazole (256 µg/ml), flucytosine (32 µg/ml), and ketoconazole (32 µg/ml). Strains in bold font were also tested in mice and sequenced.
(TIFF)
