# Supplementary figures and images for: Cryptococcus gattii VGIII Isolates Causing Infections in HIV/AIDS Patients in Southern California: Identification of the Local Environmental Source as Arboreal
Source: PLoS Pathog. 2014 Aug 21;10(8):e1004285. doi: 10.1371/journal.ppat.1004285 (PMC4140843; doi:10.1371/journal.ppat.1004285)

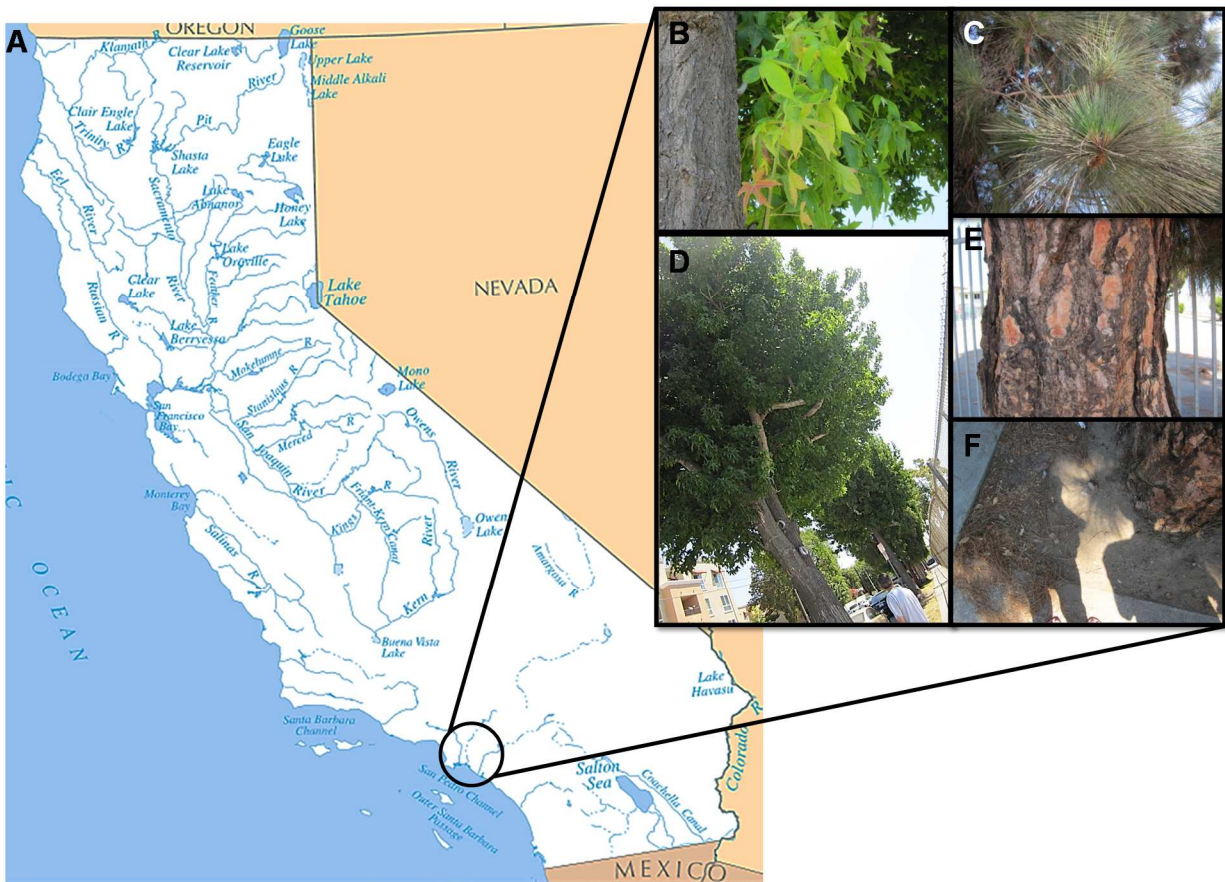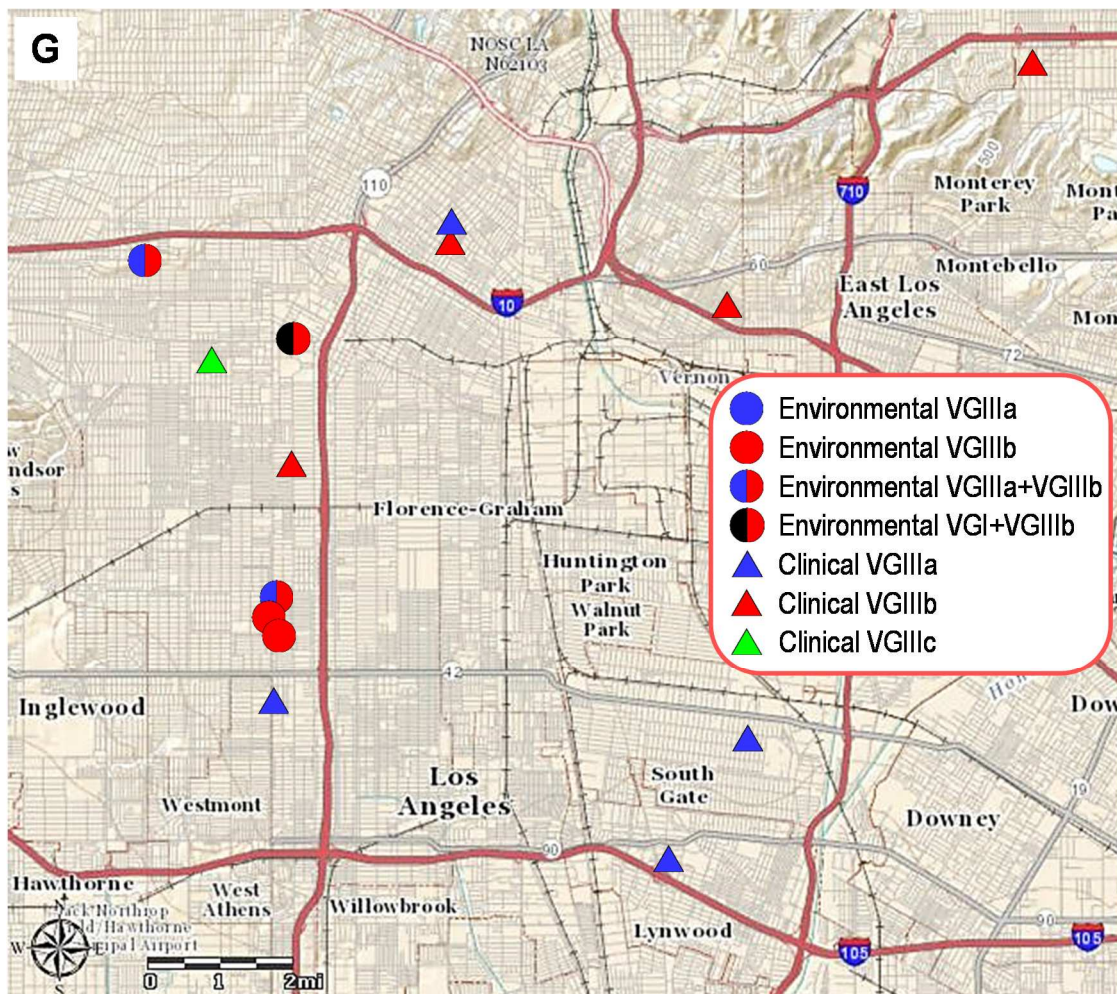

Supplement: Figure S1 — Geographic sampling area. (A) Swab and soil samples collected from 24 locations throughout the greater Los Angeles area, California, USA (Black circle).109 trees and 58 soil samples were obtained. Map data NationalAtlas.gov. (B–F) C. gattii environmental isolates were associated with non-Eucalyptus hosts. (B, C) 78-1-S3A was isolated from swab samples of Liquidambar styraciflua (American sweet gum) and MCP-1A (D, E, F) was isolated from soil samples of Pinus canariensis (Canary Island pine). Locations of positive environmental samples and residences of known clinical infections (G). Locations of known clinical cases noted with triangles and environmental isolates with circles. Different colored markers indicate molecular types associated with the particular location Blue = VGIIIb, Red VGIIIa, and green = VGIIIc. Map data NationalAtlas.gov. (PDF) [file ppat.1004285.s001.pdf]

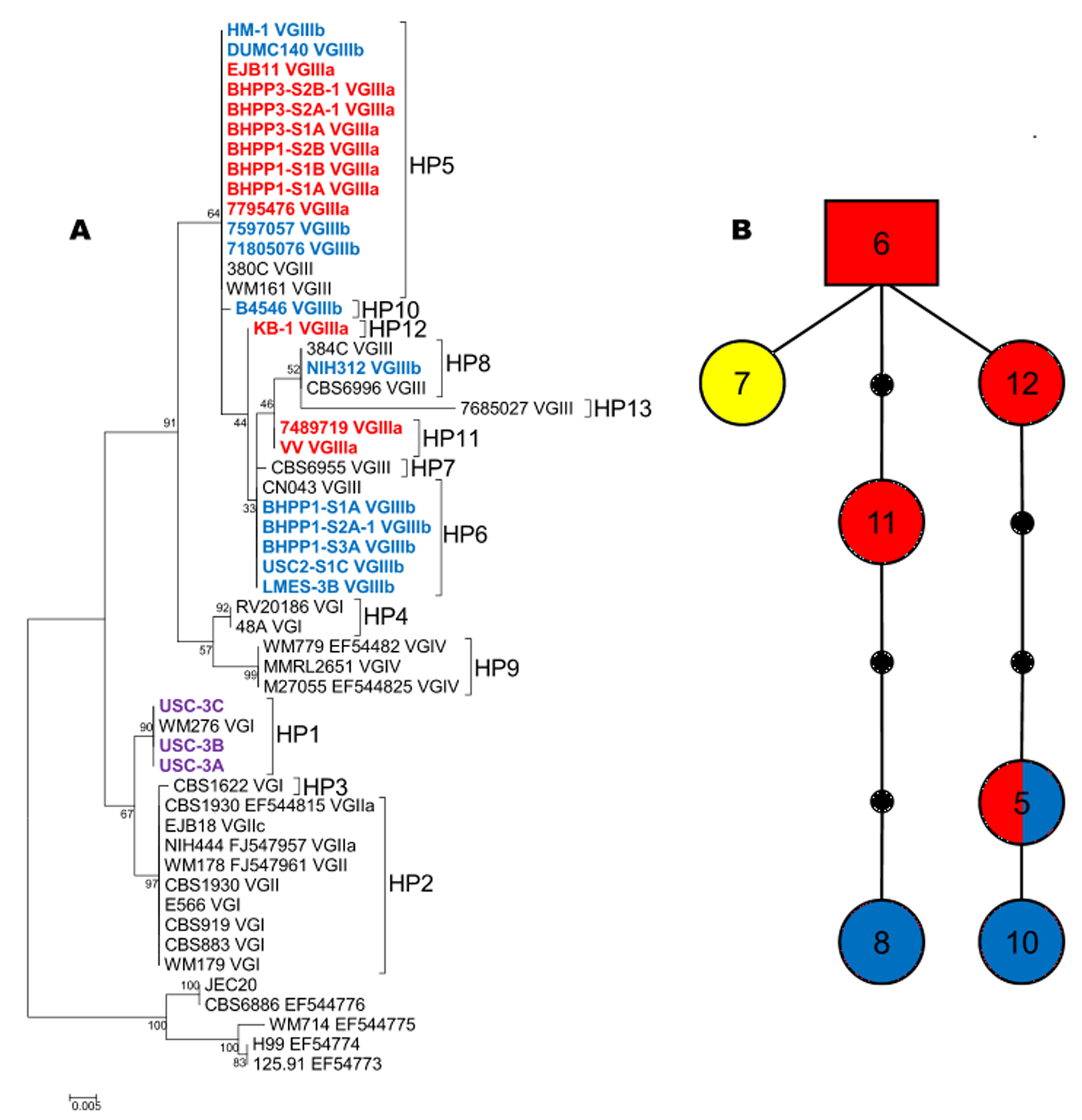

Supplement: Figure S2 — Phylogenetic and haplotype network analysis of ATP6 sequences. (A) Phylogenetic tree for ATP6 using the Maximum Likelihood (MLC) method based on Tamura-Nei model applying Neighbor-Join and 500 bootstrap replicates. The 500 bootstrap tree with the highest log likelihood is shown. Analysis involved 53 nucleotide sequences and 612 positions. Scale bar indicated 0.005 substitutions per nucleotide. Mitochondrial Haplotype (HP) allele number consistent with Bovers et al. 2009. (B) Haplotype network analysis of ATP6 sequences. Alleles are colored red representing VGIIIa, blue representing VGIIIb, and dual-coloration representing alleles shared between the VGIIIa and VGIIIb molecular types. Alleles in squares represent the proposed ancestral allele, circles represent alleles present in the population, lines between alleles represent one predicted evolutionary event, and smaller black circles represent alleles that have not been recovered, or which may no longer be represented in the population. (TIF) [file ppat.1004285.s002.tif]

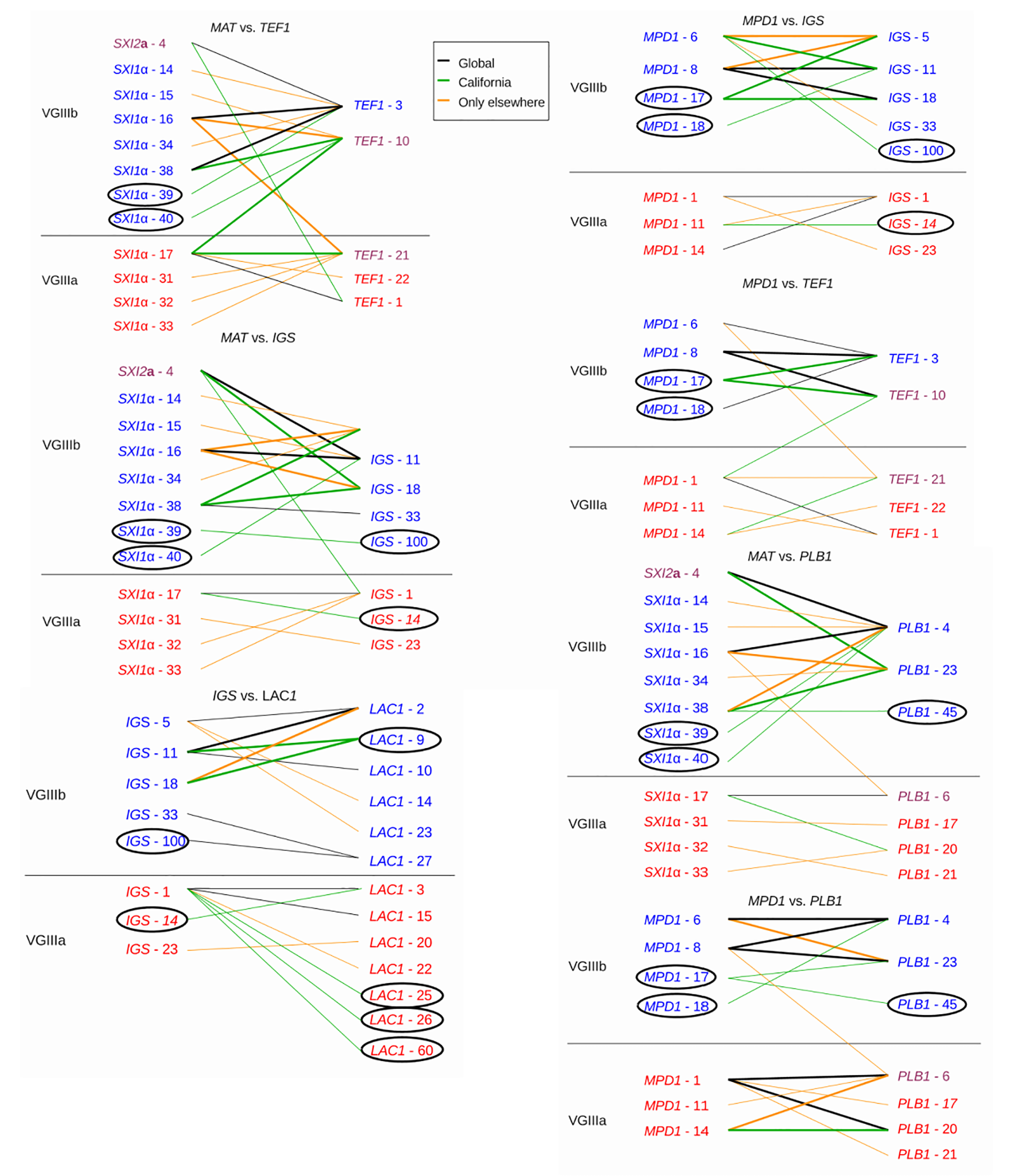

Supplement: Figure S3 — Additional paired allele diagrams. Informative paired allele diagrams depicting MLST data. An hourglass shape indicates that all four allele combinations were observed (AB, ab, Ab, aB), providing evidence for recombination. Alleles unique to California are circled, while black lines indicate combinations found both in Californian isolates and those from elsewhere. Green lines indicate combinations found only in California, and orange lines indicate combinations only found outside of California. (TIF) [file ppat.1004285.s003.tif]

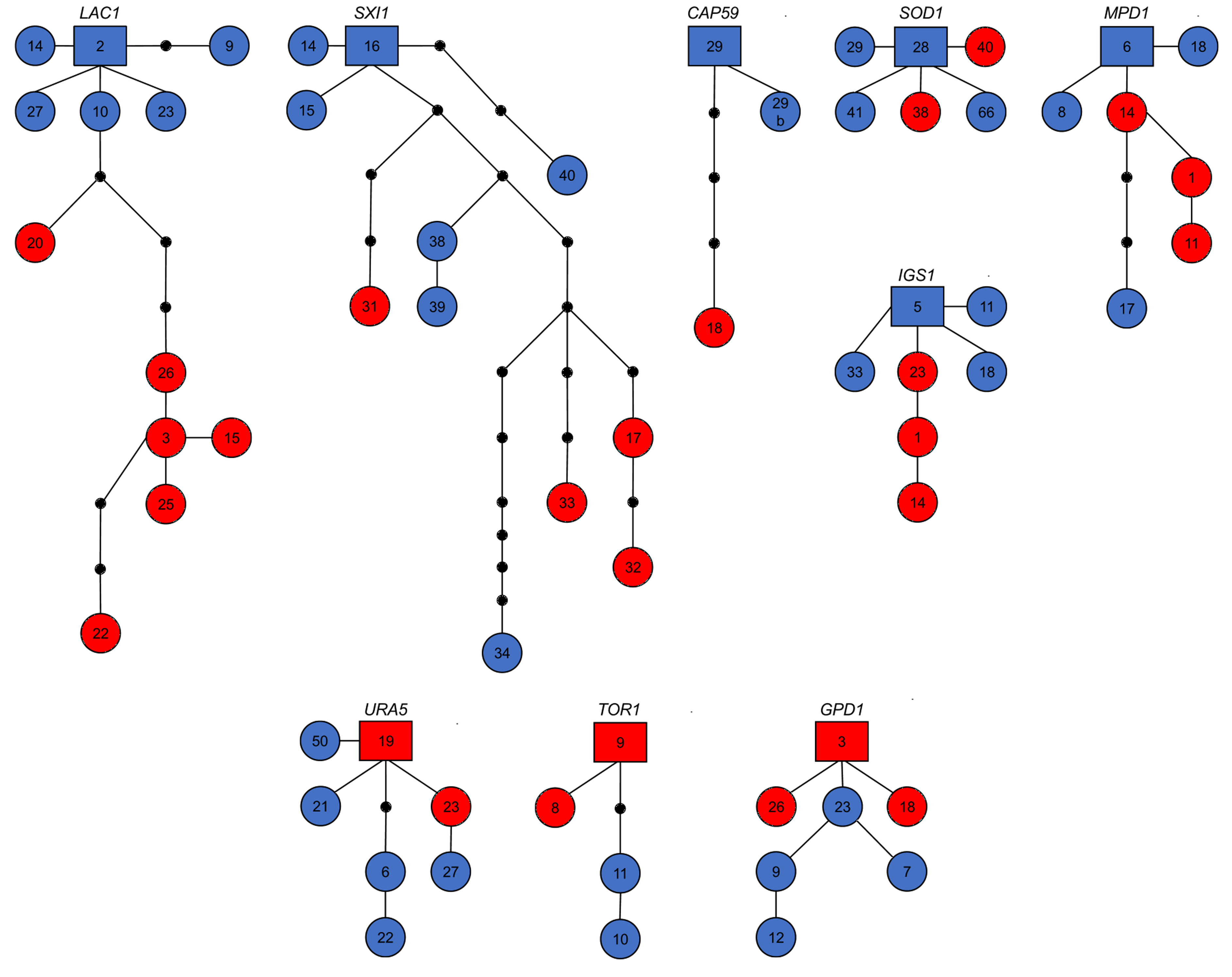

Supplement: Figure S4 — Additional haplotype networks of MLST alleles not depicted in Figure 6 depict that majority of VGIIIa and VGIIIb alleles are not shared and segregate according to molecular type. Alleles for each respective locus are indicated numerically. Alleles are colored red representing VGIIIa, blue representing VGIIIb, and dual-coloration representing alleles shared between VGIIIa and VGIIIb molecular types. Squared alleles represent the proposed ancestral allele, circles represent alleles present in the population, lines between alleles represent one predicted evolutionary event, and smaller black circles represent alleles that have not been recovered, or which may no longer be represented in the population. Haplotype analysis of the majority of MLST loci (LAC1, SXI1, CAP59, SOD1, MPD1 and IGS1) designate VGIIIb alleles as ancestral in comparison to URA5, TOR1, and GPD1 for which the designated ancestral allele belongs to the VGIIIa population. (TIFF) [file ppat.1004285.s004.tiff]

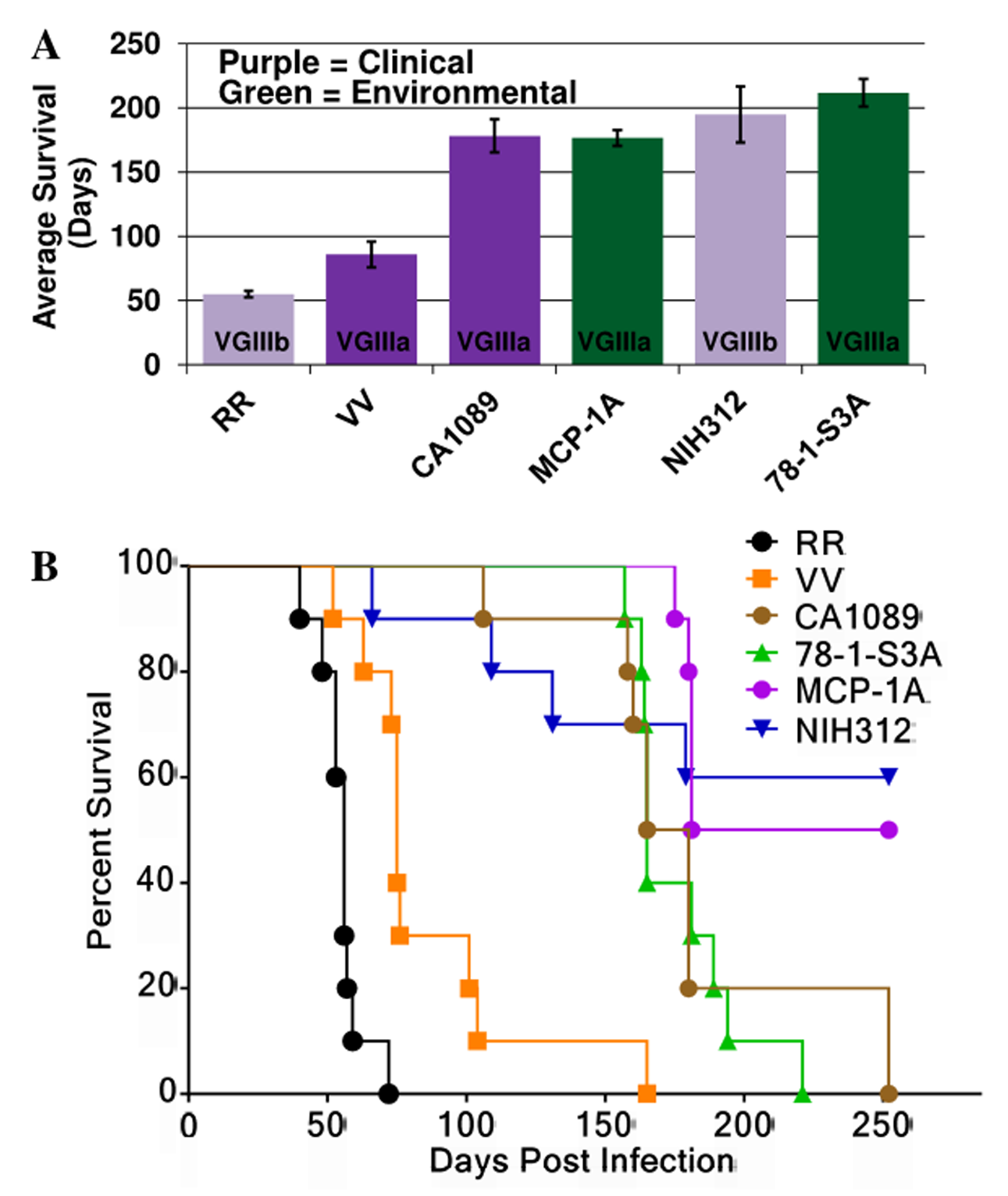

Supplement: Figure S6 — C. gattii environmental isolates are less virulent in BALB/c mice in comparison to clinical isolates. (A) Average survival time of two Californian environmental isolates in BALB/c mice in comparison to previously reported strains NIH312, RR, and VV. Clinical isolates (VGIIIa dark-purple; VGIIIb light-purple), environmental isolates (VGIIIa dark-green), and VGIIIb reference strain NIH312 (black). Average survival +/− SEM is plotted. (B)The Kaplan-Meier survival curves are presented. Ten male BALB/c mice per strain were intranasally infected with 106 cells and survival was recorded for 252 days. At the termination of the experiment some mice were still surviving isolate CA1089 (n = 2), NIH312 (n = 6), or 78-1-S3A (n = 2). (TIFF) [file ppat.1004285.s006.tiff]

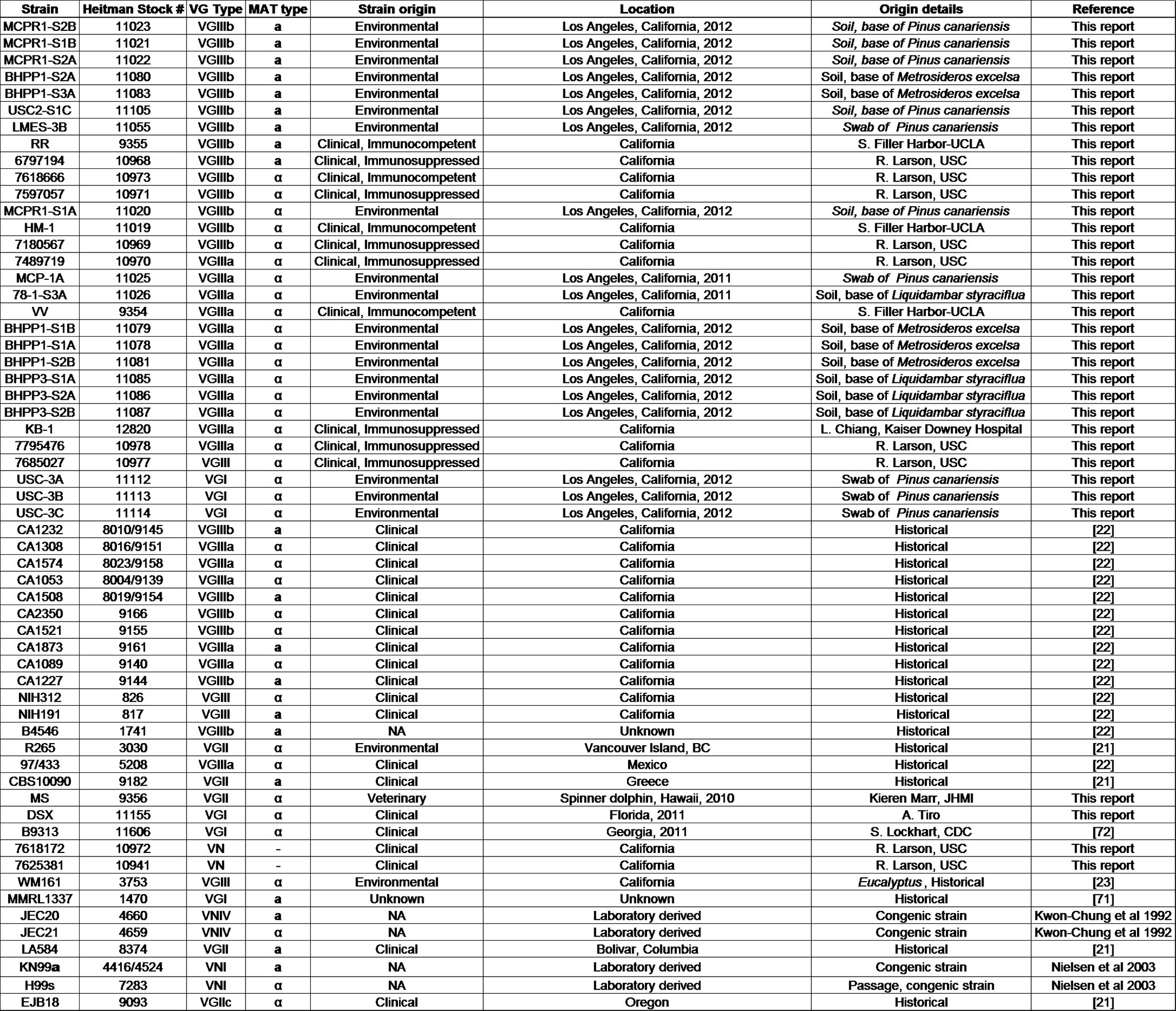

Supplement: Table S1 — Strains utilized in this report. (TIF) [file ppat.1004285.s007.tif]

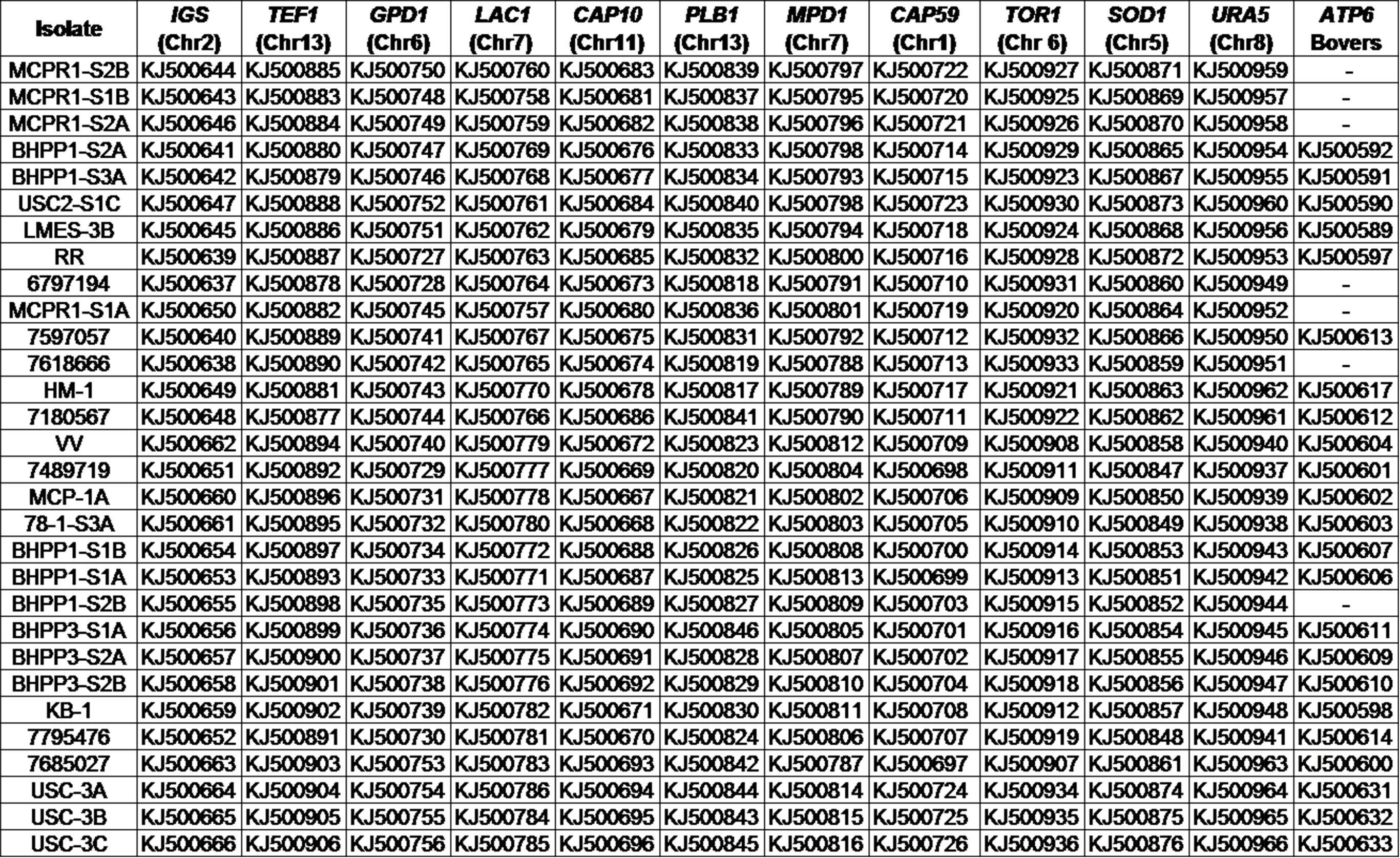

Supplement: Table S3 — List of GenBank accession numbers and allele designations for all isolates sequenced in this report. (TIF) [file ppat.1004285.s009.tif]

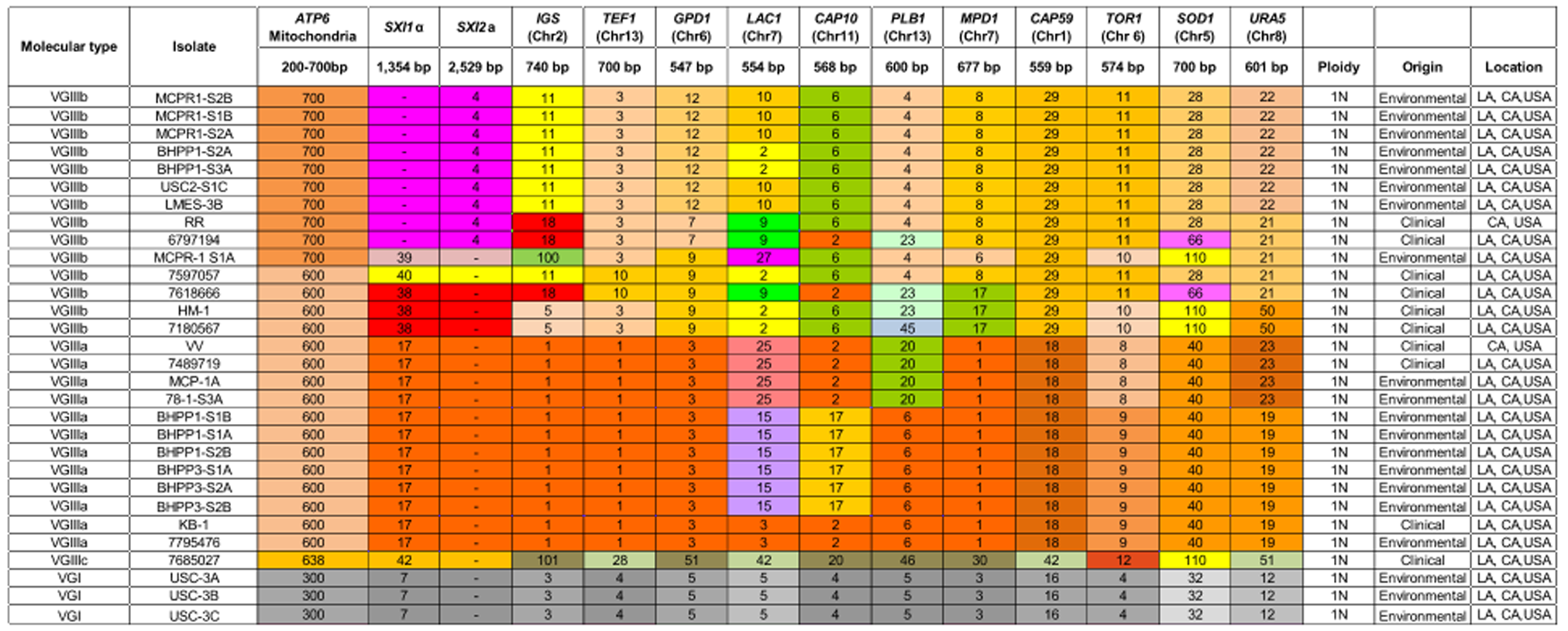

Supplement: Table S4 — Extended MLST table including ploidy and ATP6 PCR product length of newly identified C. gattii strains. Multilocus sequence typing was performed on 4 additional MLST loci (CAP59, TOR1, SOD1, and URA5) to extend analysis to a total of 12 MLST loci. Unique alleles were assigned a distinct color for each marker. FACS was performed on all isolates utilizing standard haploid (EJB18) and diploid (XL143) controls. ATP6 PCR was amplified utilizing Primers ATP6-F and ATP6-R as described in Table S2. (TIF) [file ppat.1004285.s010.tif]

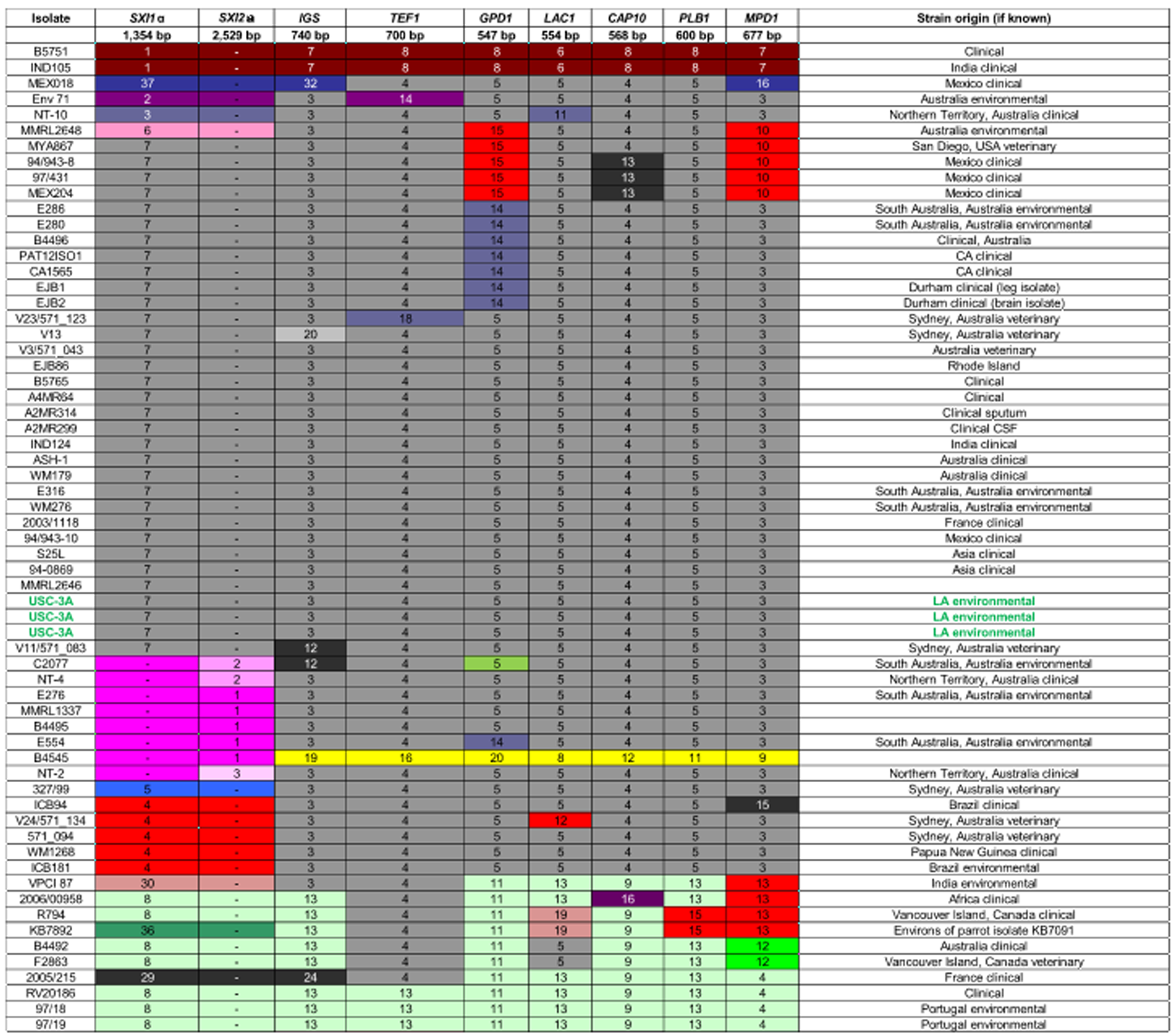

Supplement: Table S5 — Global molecular analysis of C. gattii VGI isolates endemic to Southern California. Multilocus sequence typing was performed on 8 loci. Unique alleles were assigned a distinct color for each marker. New environmental (green, n = 3) VGI isolates from California are compared to previously analyzed VGI isolates. (TIFF) [file ppat.1004285.s011.tiff]

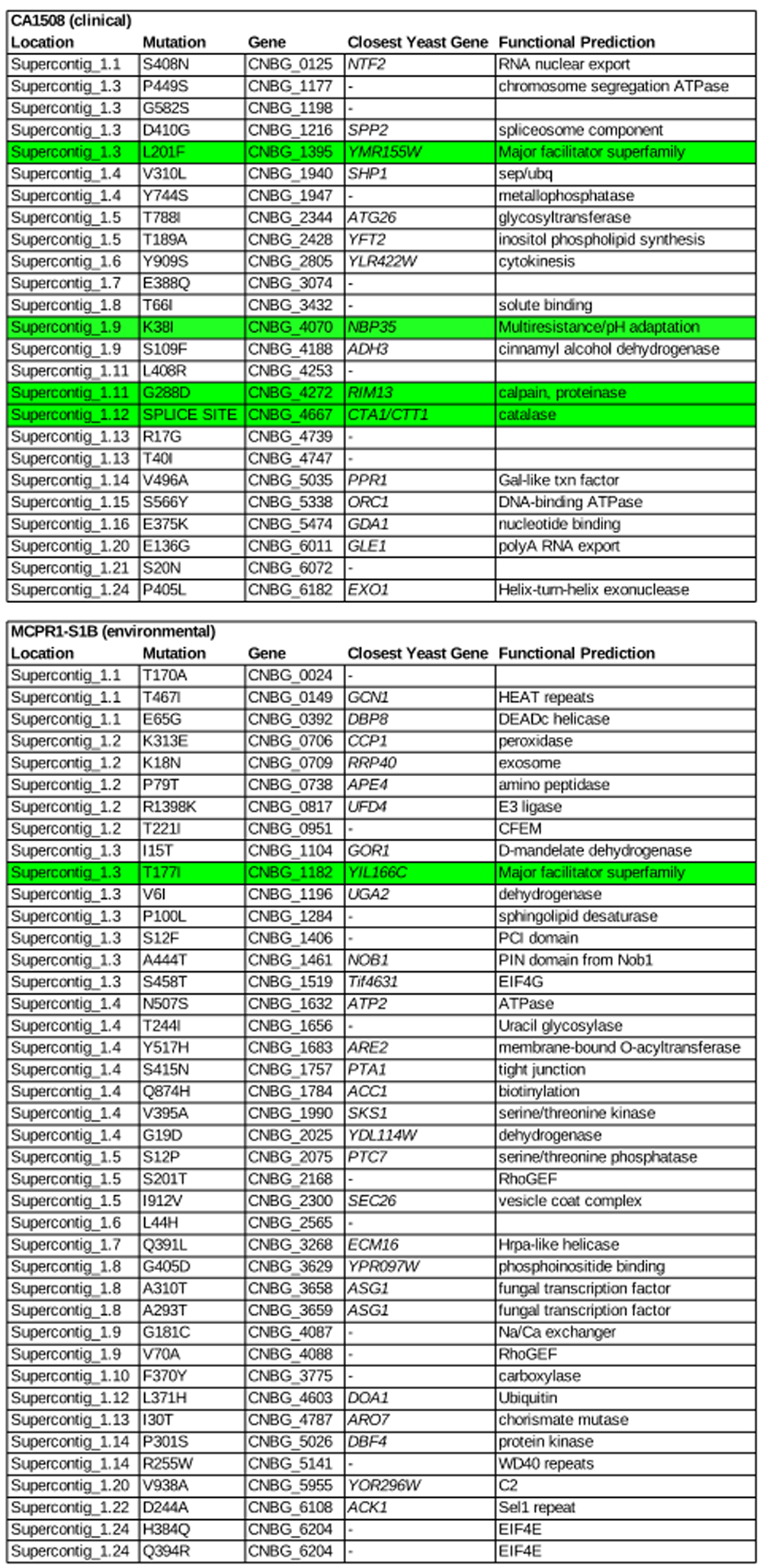

Supplement: Table S6 — SNPs observed from whole genome sequencing of MCPR1-S1A and CA1508. Whole genome sequencing of MLST matched VGIIIa group 1 pairs of environmental and clinical isolates. (TIFF) [file ppat.1004285.s012.tiff]

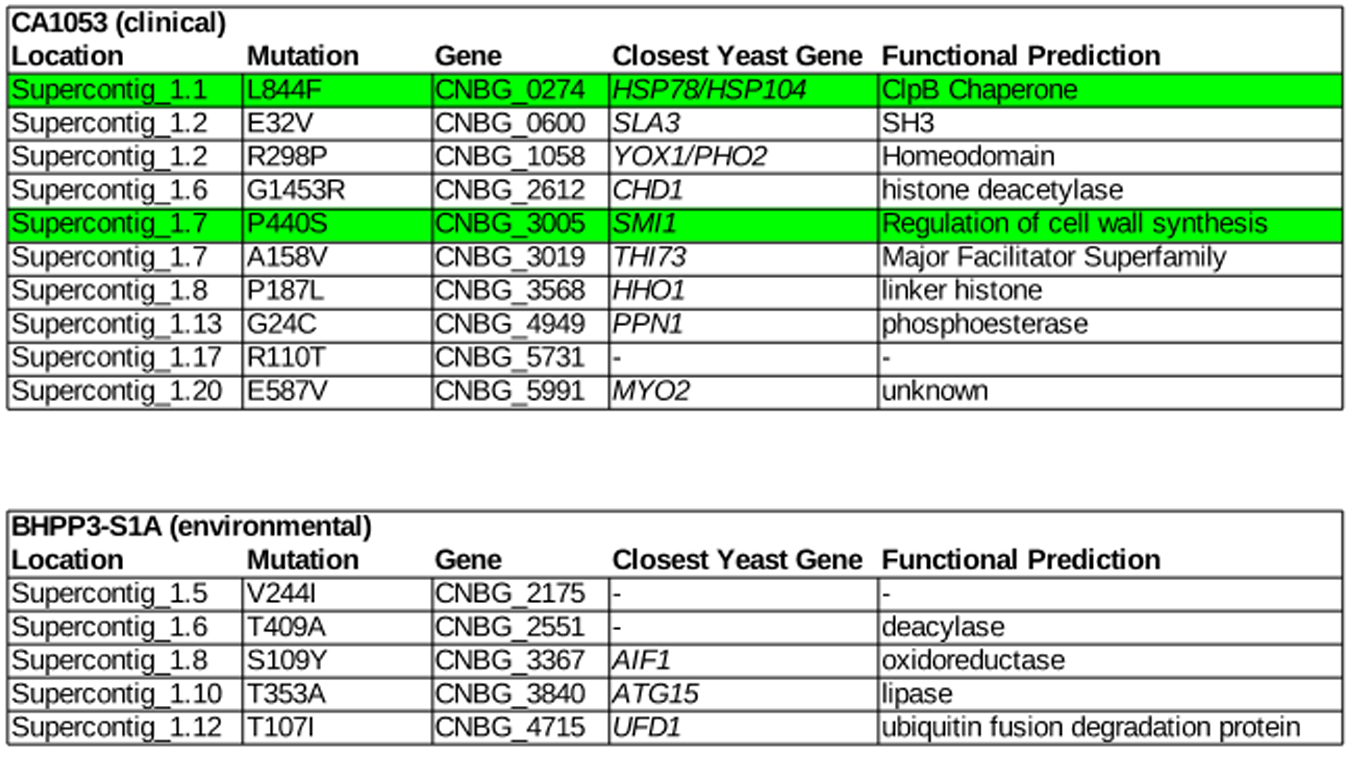

Supplement: Table S7 — SNPs observed from whole genome sequencing of BHPP3-S1A and CA1053. Whole genome sequencing of MLST matched VGIIIa group 2 pairs of environmental and clinical isolates. (TIFF) [file ppat.1004285.s013.tiff]

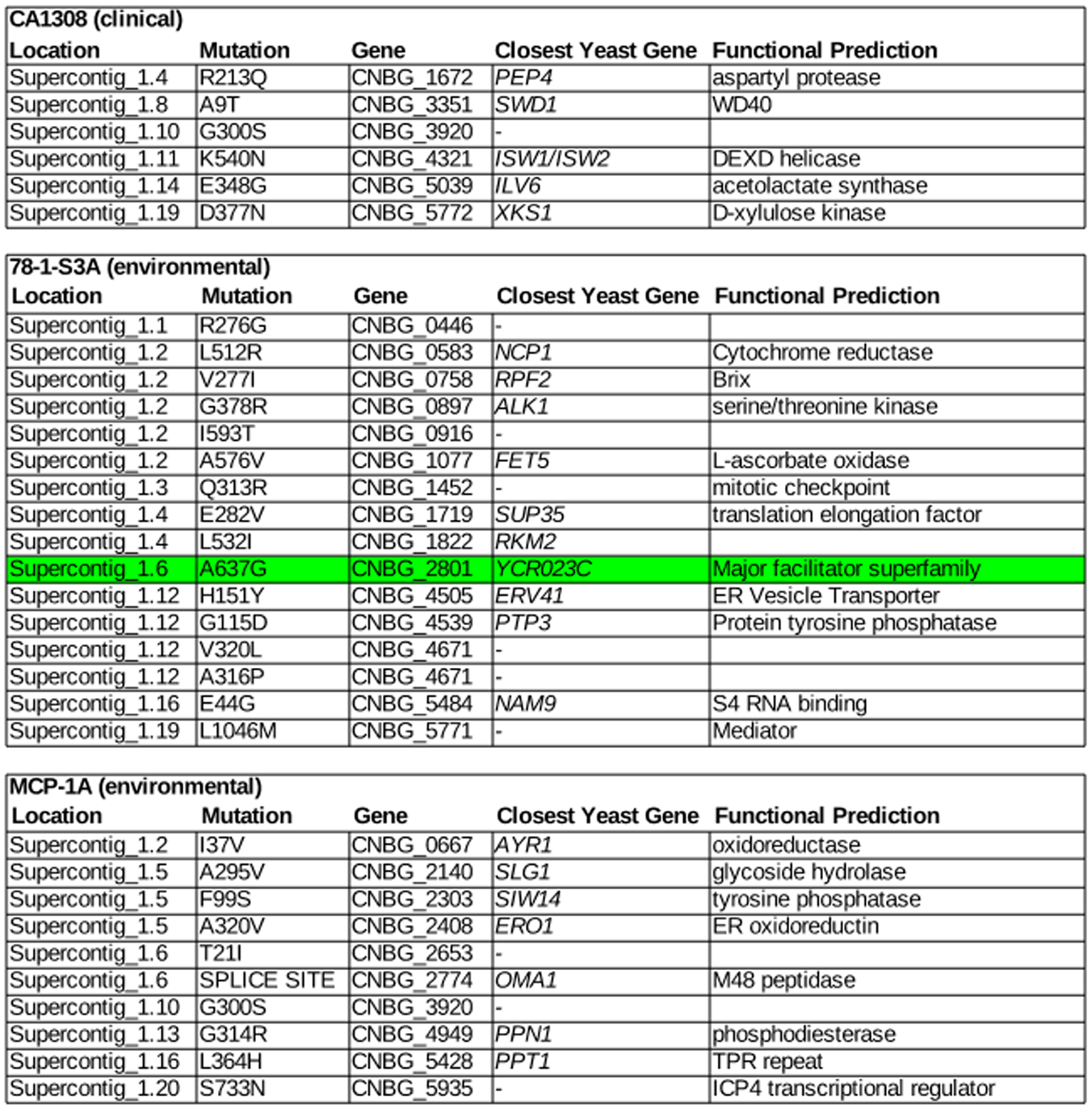

Supplement: Table S8 — SNPs observed from whole genome sequencing of 78-1-S3A, MCP-1A, and CA1308. Whole genome sequencing of MLST matched trio of VGIIIb environmental and clinical isolates. (TIFF) [file ppat.1004285.s014.tiff]

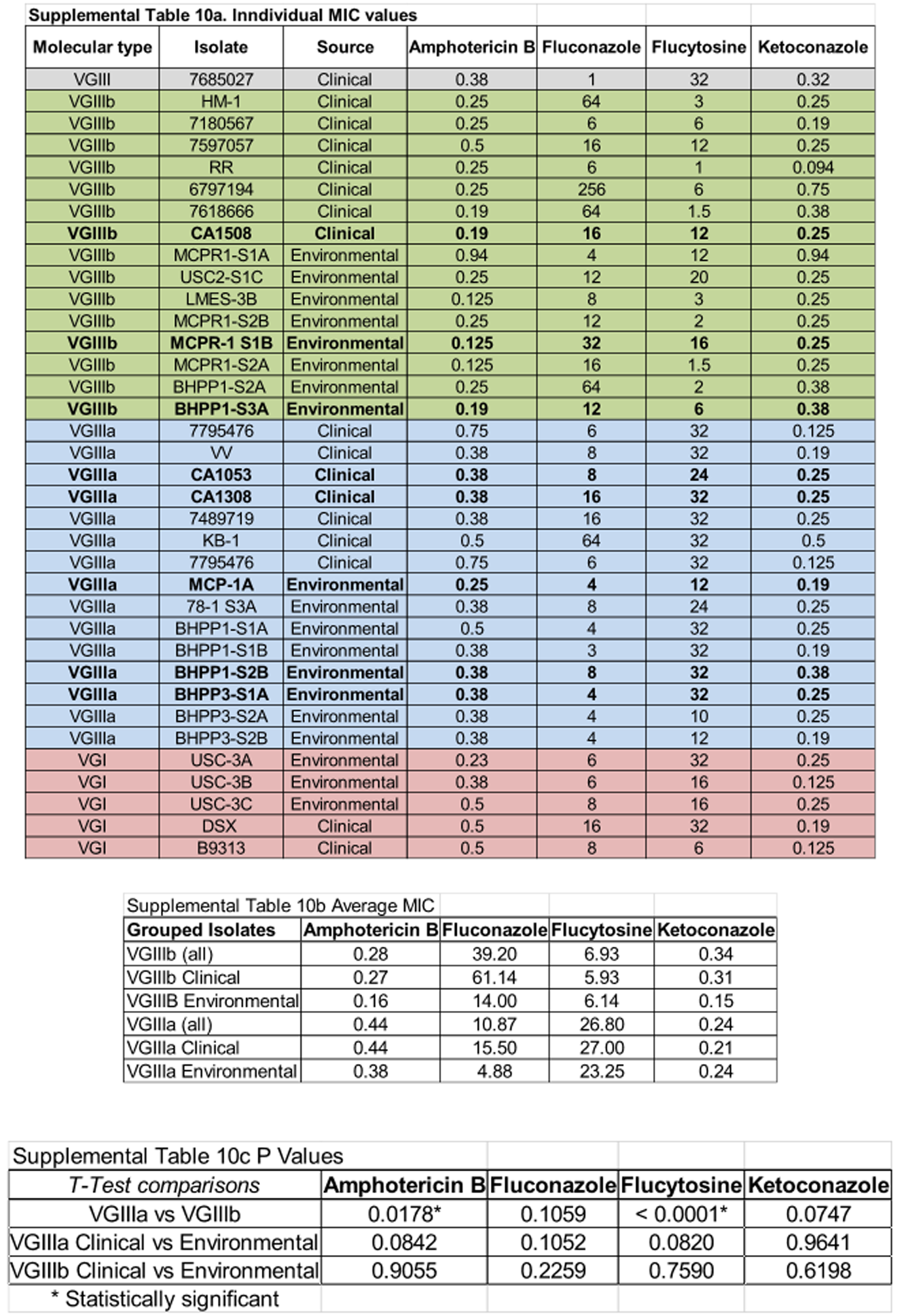

Supplement: Table S10 — Antifungal sensitivity of C. gattii isolates. (A) VGIIIa and VGIIIb MIC µg/ml results for C. gattii isolates as determined by Etest. (B) Average MIC values of VGIIIa, VGIIIb, clinical and environmental isolates. (C) P values calculated by GraphPad Prism version 6.03 for each group. Maximum concentrations of Etest were amphotericin B (32 µg/ml), fluconazole (256 µg/ml), flucytosine (32 µg/ml), and ketoconazole (32 µg/ml). Strains in bold font were also tested in mice and sequenced. (TIFF) [file ppat.1004285.s016.tiff]
